# Exploring Patient Perspectives, Engagement, and Output Quality in Doctor-Supervised Use of Artificial Intelligence During Informed Consent Consultation With ChatGPT and Retrieval Augmented Generation (RAG): Quantitative Exploratory Study

**DOI:** 10.2196/73717

**Published:** 2025-10-22

**Authors:** Sascha Donner, Poroshista Knauer, Arne Kienzle, Jesse Dinneen, Joost Burger, Carsten Perka, Stefanie Donner

**Affiliations:** 1 Berlin School of Library and Information Science Humboldt-Universität zu Berlin Berlin Germany; 2 Center for Musculoskeletal Surgery Charité - Universitätsmedizin Berlin Berlin Germany; 3 Swedish School of Library and Information Science University of Borås Borås Sweden

**Keywords:** large language model, GPT-4, AI chatbot, hallucinations, perioperative management, total hip arthroplasty, patient education

## Abstract

**Background:**

Comprehensive preoperative education is essential for optimizing outcomes and ensuring informed consent in patients undergoing total hip arthroplasty (THA). Emerging artificial intelligence (AI) tools, such as ChatGPT, offer scalable support for patient education, but their clinical application requires rigorous evaluation to ensure accuracy, safety, and trust.

**Objective:**

This study assessed patients’ preferences and satisfaction with AI-assisted informed consent in THA, comparing traditional physician consultations to those supported by native ChatGPT and a customized version enhanced with retrieval-augmented generation (RAG). It also examined how state anxiety and general attitudes toward AI affect preferences for AI-supported consent and whether RAG integration improves ChatGPT response quality.

**Methods:**

A total of 36 patients scheduled for elective THA were assigned to one of three groups (12 each): (1) standard physician-only consultations (control), (2) physician-assisted consultations supported by native ChatGPT, and (3) supported by ChatGPT enhanced through RAG. Data collection involved standardized Likert scale questionnaires assessing patient satisfaction with the consent process, perceived informedness, anxiety levels, and attitudes toward AI. The ChatGPT responses were independently evaluated by physicians for relevance, accuracy, clarity, completeness, adherence to evidence-based guidelines, and appropriate length. Instances of hallucinations, factually incorrect or misleading outputs, were identified and rated by severity. Statistical analyses compared outcomes across groups and explored associations.

**Results:**

Patients interacting with the ChatGPT+RAG model reported significantly higher satisfaction levels with information delivery (*P*=.01) and perceived level of informedness (*P*=.01) than those using the native ChatGPT model. The mean number of patient questions in the control group was 20, compared with 39 in the native ChatGPT group (*P*=.06) and 52 in the ChatGPT+RAG group (*P*=.002). The majority of participants across all groups preferred a human clinician providing less accurate information over a more accurate AI-only assistant. These preferences were not influenced by sociodemographic variables (age, gender, and education), health literacy, state anxiety, or general attitudes toward AI. The ChatGPT+RAG model outperformed the native ChatGPT model across all evaluated response quality dimensions (all *P*<.01) and exhibited a significantly lower hallucination rate (5/52, 10% versus 15/39, 38%; *P*=.002).

**Conclusions:**

Integrating RAG with ChatGPT significantly improves the quality, clarity, and reliability of preoperative information, enhancing patient satisfaction and engagement beyond native ChatGPT. However, patients maintain a strong preference for physician-led informed consent, underscoring the role of AI chatbots as complementary tools rather than replacements. These findings support the cautious adoption of customized AI assistants to augment, not substitute, human interaction in surgical consent processes.

## Introduction

It is legally and ethically mandatory to provide patients with adequate information during a preoperative surgical consent consultation. Preoperative education serves not only as a vital communication tool between physician and patient but also holds forensic importance. Time constraints in surgical practice often limit the amount of time surgeons can dedicate to patients to inform them about the surgical procedure, risks, and postoperative care during the informed consent procedure. This can leave patients feeling underinformed and anxious about their upcoming surgery. With increasing constraints on medical staff resources and growing individual workloads, there is also an increasing interest in boosting efficiency, particularly using artificial intelligence (AI) [1,2].

Recent technological advancements have led to the development of large language model-based tools such as the AI chatbot ChatGPT, which demonstrate improved text analysis and generation capabilities that have the potential to greatly enhance patient education and outcomes [3-6]. Patients can ask specific questions regarding their planned surgical procedure and perioperative management and get in return answers relevant to their questions with a possibly high level of accuracy. The potential of AI, especially AI chatbots powered by generative pretrained transformers (GPTs) [7,8], is increasingly recognized for its ability to transform patient engagement [9]. However, the use of those tools also carries potential risks. Notably, the tools can produce inaccurate, misleading, and potentially harmful responses, often referred to as hallucinations [10]. Such hallucinations are expected to decrease with more advanced versions like GPT-4o [11]. Particularly promising is the use of methods such as retrieval-augmented generation (RAG), where the AI chatbot is provided with a database containing additional, subject-specific information, though the effects of such an approach on responses to patients’ questions have not yet been examined. Recent findings confirm that GPT-4 and GPT-4o exhibit exceptional accuracy and efficiency in language and reasoning capabilities [12-15]. However, they also emphasize the importance of developing more comprehensive benchmarks and robust evaluation frameworks, incorporating qualitative assessments, human judgment, and detailed error analysis [16,17]. This study, therefore, also aims at investigating prompt-based assessment in the informed consent procedure.

Given its high degree of standardization and the development of robust postoperative care protocols in recent years, hip arthroplasty serves as an exemplary model for exploring the integration of AI into clinical procedures and patient education frameworks [18-20]. Total hip arthroplasty (THA) is a procedure where a damaged or worn-out hip joint is replaced with an artificial joint, particularly for patients with hip osteoarthritis. This operation, widely regarded as the “operation of the century,” is noted for its profound impact on pain reduction and the enhancement of patients’ quality of life [21]. Before the operation, patients usually have questions concerning the procedure and the physical rehabilitation process [22,23].

The process of information delivery is highly important and represents a central pillar of patient education and the reduction of fear and anxiety [24,25]. Preoperative anxiety is a common experience among patients, often stemming from inadequate information about the surgical process. Therefore, patient education is of critical importance prior to surgery. Evidence supports that better-prepared patients—those who receive thorough preoperative education and physical conditioning—exhibit improved postoperative outcomes, including accelerated recovery and reduced complications. Therefore, enhanced patient education is an important component of surgical care [26].

This is the first study to investigate surgical informed consent consultation using AI chatbots prior to THA. Here, we analyzed ChatGPT’s responses to patients’ posed questions preceding THA to assess its potential in preoperative patient education. Beyond the immediate benefits of patient education, the study explores the quality of the responses and potential hallucinations outputted by ChatGPT and systematically scrutinizes the system’s current ability to deliver adequate responses. This study also examines patients’ attitudes toward the use of an AI chatbot in a preoperative setting, along with potential cofactors influencing these attitudes. Assessing patient satisfaction with AI-driven consultations offers valuable insights into the acceptance and effectiveness of AI in patient interactions. The findings can inform the design of improved systems and the development of guidelines and protocols for integrating AI into patient care, particularly in sensitive areas like surgical informed consent.

Research questions:

1. What are patients’ preferences regarding the use of an AI chatbot in conjunction with a physician in surgical informed consent, in comparison to a physician only?
2. How does patient satisfaction compare across informed consent procedures involving only a physician, a physician and ChatGPT, or a physician and RAG-integrated ChatGPT?
3. How does the integration of RAG with ChatGPT improve the quality of responses to patients’ questions compared with the native ChatGPT model?
4. How do patient sociodemographic characteristics and general attitude toward AI influence patients’ preference toward using an AI chatbot in surgical informed consent?

## Methods

### ChatGPT

In this study, we used the OpenAI ChatGPT version 4o (May 13, 2024). To ensure consistency, all questions were presented to ChatGPT in a single, continuous chat session (per patient). The questions were asked by patients during their preoperative surgical consultation before THA in our clinical practice. For the purposes of the study, all patient queries were transcribed verbatim by the doctor into the ChatGPT interface.

In our study, we have used ChatGPT in two ways: (1) without any customization (native) and (2) with customization via RAG, described further below. The second approach allowed ChatGPT to access and incorporate subject-specific knowledge and thus potentially enhance the relevancy and accuracy of its responses. The performance and output accuracy of the 2 systems were compared as described below.

No patient-specific data was entered. The study was conducted in Germany, with all interactions and materials presented exclusively in German.

### Recruitment

Patients scheduled for elective, primary THA were recruited from the orthopedic outpatient clinic or on the day of the information session. The study was conducted from May to July 2024. The consent consultations were conducted by 2 physicians who were pretrained based on the prepared frequently asked questions (FAQ) documentation.

Inclusion Criteria:

1. Age: Participants older than 18 years.
2. Diagnosis: Diagnosed with primary hip arthritis.
3. Treatment History: Currently indicated for primary hip arthroplasty.
4. Language: Able to read, understand, and complete study-related questionnaires in German.

Exclusion Criteria:

1. Cognitive impairment: Any cognitive impairment or psychiatric condition that would hinder the participant’s ability to provide informed consent and comply with study procedures.
2. Prior operation: History of primary or revision arthroplasty on the contralateral side.

The participants were provided with detailed written information about the aim of the study, the study procedure, the inclusion and exclusion criteria, and the informed consent about their participation in the study. The participants had the opportunity to ask questions about any aspect of the information provided, the questionnaires, and the study procedure.

### Study Arms

Each patient was assigned to one of the following study arms:

1. Control group (human only): A physician conducting an informed consent consultation in a traditional face-to-face setting.
2. Intervention group I (native ChatGPT): ChatGPT was used in the questions and answers (Q&A) session in the presence of a physician.
3. Intervention group II (ChatGPT+RAG): ChatGPT+RAG was used in the Q&A session in the presence of a physician.

From the total population of 36 patients, the first 12 patients were assigned to the Control Group. The next 12 patients were included in the native ChatGPT group, and the final 12 patients were allocated to the ChatGPT+RAG group. All 36 patients enrolled in the study completed the full informed consent procedure and associated assessments, with no dropouts or exclusions.

In the RAG group, an extensive internal document containing all the information about the surgical procedure and the perioperative management at our hospital was compiled and integrated into ChatGPT via the RAG approach. This integration was achieved through the GPT app Keymate.AI (Keymate AI, Inc), which adds the information from the document into the model’s context window, effectively functioning as short-term memory. In the intervention group I, we added the following prompt text: “Please provide responses only in German and in the style of a medical doctor” (Bitte antworte nur in deutscher Sprache und im Stil einer Ärztin oder eines Arztes). In the intervention group II, we added a prompt text prior to each question posed to the AI chatbot: “Please provide responses only in German and in the style of a medical doctor. Please draw only on our internal FAQ document when responding” (Bitte antworte nur in deutscher Sprache und im Stil einer Ärztin oder eines Arztes. Bitte nutze zur Beantwortung ausschließlich die Informationen, die in unserem internen FAQ-Dokument enthalten sind). This setup is supposed to ensure that the AI chatbot responds preferably to the specific clinical information provided.

### Data Collection Methods

#### Patient Sociodemographic Questionnaire and Class-Index

We collected the following patients’ characteristics: age, gender, and highest level of education.

#### Patients’ Preferences and Satisfaction Questionnaire

Data were collected using a self-constructed questionnaire that included 5 questions on a 7-point Likert scale regarding the preference of a physician in the informed consent procedure, the level of satisfaction with informedness, the procedure of information transfer, the quantity of information, as well as the preference for having a physician even with less information. Patients could also express their need for more information (yes/no).

#### Health Literacy Test for Total Hip Arthroplasty

To ensure that patients were capable of giving informed consent and could, in principle, understand the outputs of the AI chatbots, patients’ health literacy was measured with a questionnaire consisting of 9 multiple-choice questions. This questionnaire was adapted from a questionnaire for total knee arthroplasty [27], but with knee information and questions replaced by those for hip arthroplasty.

#### The General Attitudes Towards Artificial Intelligence Scale

The General Attitudes towards Artificial Intelligence Scale (GAAIS) introduced by Schepman and Rodway [28] was evaluated to assess patients’ attitudes toward AI. This 20-question scale is divided into a positive (12 questions) and a negative (8 questions) subscale. Both subscales capture emotions in line with their value. The 5-item scale ranges from 1 to 5. The mean of the positive questions constitutes the overall score for the positive subscale, while the same approach is applied to calculate the overall score for the negative subscale. The higher the score, the more positive the attitude toward AI. An overall scale mean was not recommended.

#### State-Trait Anxiety Inventory

To assess the actual state of anxiety (influenced by the current situation) and trait anxiety (the general anxiety of the patient), the Spielberger State-Trait Anxiety Inventory [29] was administered, specifically Grimm’s abbreviated, validated version [30].

#### Output Evaluation of AI Chatbot Responses

To assess the quality of the AI chatbot responses, we conducted a detailed evaluation of the questions asked by patients during their interactions with the AI chatbot and the model’s responses.

First, ChatGPT’s output was evaluated based on Magruder et al [31] for relevance, accuracy, clarity, completeness, and evidence-based. Each criterion ranges from 1 to 5, with 5 being the best answer. We did not rate consistency between questions since every question was posed only once to the AI chatbot. For the accuracy criterion, we compared the responses with the official guideline on indication criteria for total hip arthritis of the “German Society for Orthopedics and Trauma” [18], the FAQs of the German Arthroplasty Association [32], and our internal standard operating procedures. To evaluate the length of ChatGPT’s output, we introduced an additional criterion that holds the balance between succinctness and informativeness. This criterion focused on whether the response was appropriately concise while fully addressing the question without unnecessary elaboration. Scores ranged from 1 (overly brief or excessively long, missing necessary information, or including redundant content) to 5 (ideal length, providing a complete and focused answer that matched the question).

Second, we evaluated the level to which the AI chatbot produced so-called hallucinations, that is, “content that is either nonsensical or unfaithful to the provided source content” [33,34]. ChatGPT’s output was classified as fully correct, partly hallucinated, and fully hallucinated. In addition, we annotated the level of hallucination using 3 degrees, following Rawte [35] and adapted for the evaluation of AI chatbot outputs in the medical field. The degrees are mild, moderate, and alarming, with mild indicating minor hallucination that is superficial in terms of its impact, moderate indicating a level of hallucination that introduces facts that are either fictitious or tangential to the topic at hand and which could cause the patient to become confused or feel insecure, and alarming indicating added information pieces that bear a radical dissemblance from the topic fed via the prompt or which could lead to serious harm to the patient.

We have added all questionnaires used for data collection as Multimedia Appendix 1 (study questionnaires).

### Procedure

**Figure 1:**
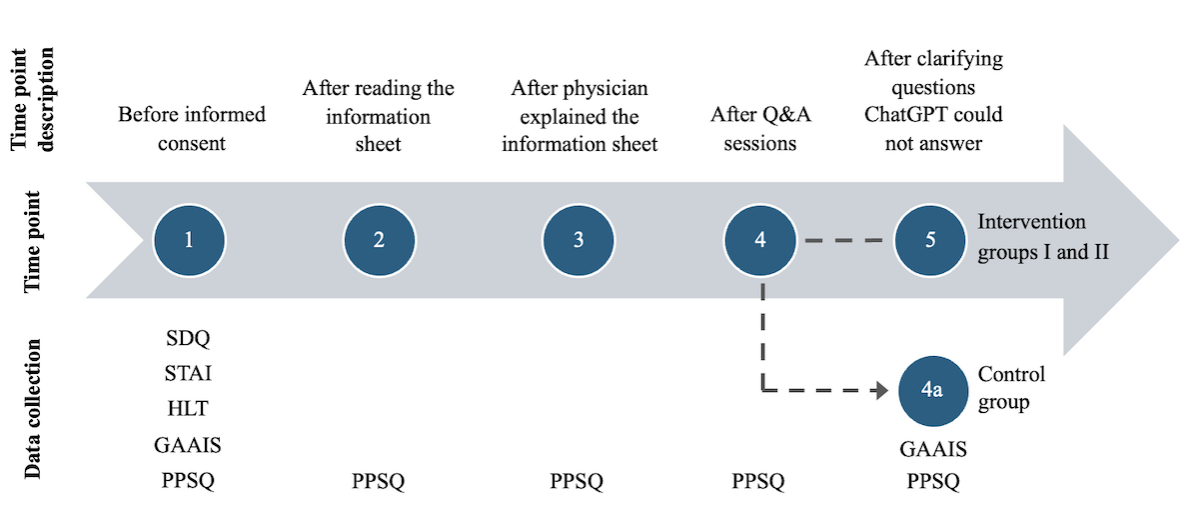
Study procedure and time points of data collection. GAAIS: General Attitudes towards Artificial Intelligence Scale; HLT: health literacy test; PPSQ: patients’ preferences and satisfaction questionnaire; Q&A: questions and answers; SDQ: sociodemographic questionnaire; STAI: State-Trait Anxiety Inventory.

The study procedure and timing of data collection were identical for the control group and the 2 intervention groups up to time point 3, as follows (summarized in Figure 1 below).

- Baseline data collection (time point 1): Initial data were collected prior to the informed consent procedure.
- Information sheet (time point 2): After the patient had read the provided informed consent form.
- Physician explanation (time point 3): After the physician explained the operation, associated risks, and possible complications, using the information consent form as a reference. At this point in time, no questions were answered.

From time point 4 onward, the procedures diverged as follows.

- Control group (time point 4): After patients asked questions in a conventional face-to-face setting, the physician answered without AI chatbot assistance.
- Intervention groups I and II (time point 4): After patients’ questions were entered by the physician into the AI chatbot interface, and responses were read out word-for-word in German.

All hallucinated or incorrect responses were identified by the experienced physicians who were present throughout the informed consent consultations. These were explicitly addressed and corrected during the final stage of the session (time point 5), prior to concluding the informed consent process, thereby ensuring patient safety and upholding the integrity of the consultation. In other words, the final data collection step for the Control Group was time point 4, whereas the Intervention Groups had a fifth data collection step, time point 5.

This structured process ensured that all patients, regardless of their group, received clear and sufficient answers to their questions during the informed consent procedure. All Q&A of each information session were documented. All admission examinations were conducted in a standardized setting by the same orthopedic resident and were supervised by a senior orthopedic surgeon. In the following Figure 1, the course of the survey, the times of data collection, and the method are shown.

The Patients’ Preferences and Satisfaction Questionnaire (PPSQ) was completed 5 times (4 times in the human-only group) during the informed consent procedure. The GAAIS was completed at the beginning and the end of the informed consent procedure to evaluate if a change in the overall inclination toward AI has occurred during the process. The sociodemographic questionnaire, State-Trait Anxiety Inventory, and Health Literacy Test (HLT) were completed prior to the informed consent procedure.

### Statistical Methods

#### Sample Size Assumptions

In our exploratory pilot study, which was designed to generate initial insights and hypotheses for future, larger-scale investigations, a sample size of 12 participants per group (one control group and 2 intervention groups) was chosen based on the recommendations by Julious [36].

#### Data Analysis

Statistical analyses were performed using SPSS Statistics (version 29; IBM Corp).

The Shapiro-Wilk test was applied to assess the normality of the data distribution for continuous variables. The test revealed that none of the collected data were normally distributed (*P*<.05). As a result, a nonparametric statistical test was used for all analyses.

A Friedman test was conducted to analyze differences in patients’ preferences and satisfaction levels (PPSQ) across the 5 time points: at the beginning of informed consent, after reading the information sheet, after consulting the physician, after using ChatGPT, and at the end of the informed consent process. For multiple pairwise comparisons following the Friedman test, a Bonferroni correction was applied.

The Wilcoxon signed-rank test was applied to evaluate dependent pairwise differences between satisfaction levels before and after the use of an AI chatbot. The same test measured differences between the GAAIS pre- and postinformed consent.

Spearman-Rho was used to analyze the correlation between GAAIS, State, Trait, and PPSQs.

The Mann-Whitney *U* test was used to compare independent differences between groups in the sociodemographic factors, the number of questions asked by participants, the number of questions including hallucinations, and the quality of the outputs of native ChatGPT and ChatGPT+RAG.

The Kruskal-Wallis test was used to analyze the effects of the highest educational attainment on the outcomes of the patients’ preferences and satisfaction levels (PPSQ; ie, across the 7 independent variables).

Patient questions were categorized into 5 predefined groups: operation, postoperative, complication, rehabilitation, and daily life. For the analysis, patient questions from the 2 intervention groups, the native and the ChatGPT+RAG group, were distributed across these categories. The number of questions within each category was counted for both groups. The Fisher-Freeman-Halton test was used to establish significance in differences in the categorical data. Cramer V was used for the calculation of effect size for Fisher-Freeman-Halton test; Friedmans effect size was calculated via Kendall ω, r was used for effect size of Mann-Whitney *U* test, and Wilcoxon signed-rank test after the following formula:



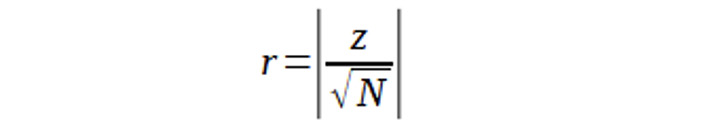



To assess interrater reliability between the investigators in assessing the AI-generated responses, Cohen κ coefficient was calculated. κ Statistic values were interpreted using the Landis and Koch criteria with values of 0.00 to 0.20 indicating slight agreement, 0.21 to 0.40 indicating fair agreement, 0.41 to 0.60 indicating moderate agreement, 0.61 to 0.80 indicating substantial agreement, and values of more than 0.80 indicating almost perfect agreement [37].

### Ethical Considerations

Approval for this prospective study was granted by the institutional review board of Charité – Universitätsmedizin Berlin (application number EA1/152/24). Informed consent was obtained from all participants prior to their inclusion in the study. No waiver of consent was requested or applied. All data collected for this study were pseudonymized prior to analysis. No personally identifiable information was included in the data. Participants did not receive any financial or material compensation for their involvement in the study. No images or supplementary materials used in this manuscript contain identifiable information of individual participants. Therefore, no additional image consent documentation was required or submitted.

## Results

### Demographic Data

Table 1 provides an overview of the sociodemographic characteristics of the study participants.

**Table 1:** Demographic characteristics of the included study participants.

			Total		Control group		Native ChatGPT		ChatGPT +RAG^a^
Patients, n			36		12		12		12
**Sex, n**									
	Male	23		5		10		8	
	Female	13		7		2		4	
**Age (years)**									
	Mean (SD)	63.2 (8.84)		65.7 (8.40)		58.3 (9.49)		65.5 (8.75)	
	Minimum	37		48		37		48	
	Maximum	83		83		77		76	
**Education, n (%)**									
	Vocational training	20 (55)		6 (50)		8 (67)		6 (50)	
	University degree	16 (45)		6 (50)		4 (33)		6 (50)	

^a^RAG: retrieval-augmented generation.

### Patients’ Preferences and Satisfaction Levels (PPSQ)

#### Need for a Human Doctor in the Informed Consent Consultation

**Figure 2:**
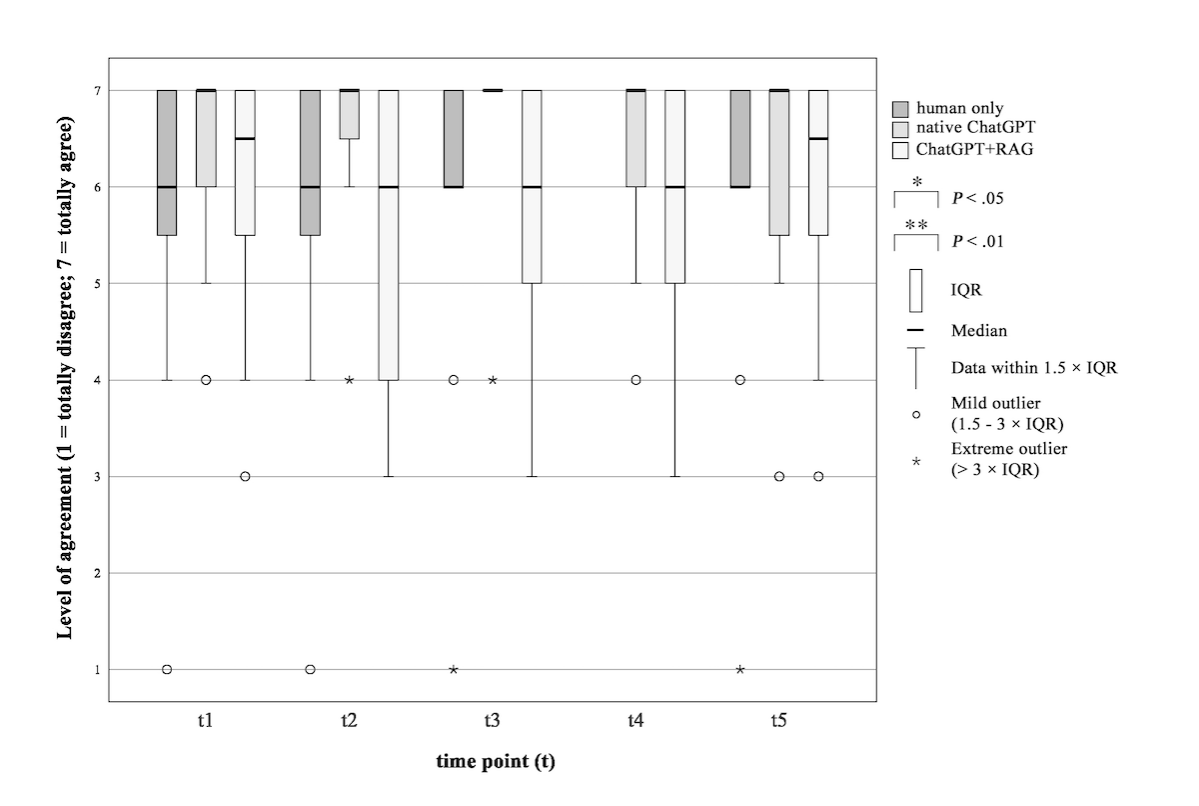
Comparison of patients’ preference for a human doctor during the surgical informed consent consultation procedure. “To what extent do you agree with the following statement: The involvement of a human doctor is essential in the surgical consent process.” RAG: retrieval-augmented generation.

Average results ranging between 5.50 and 6.65. Although slight shifts in patients’ expressed need were observed during the time points of the study procedure (Figure 2), these changes were not statistically significant. Difference of preference during time points (t) in the 3 groups: human only (*P*=.85, *χ*^2^_3_=0.818, ω=0.227); native ChatGPT (*P*=.28, *χ*^2^_4_=5.127, ω=0.107); ChatGPT+RAG (*P*=.048, *χ*^2^_4_=9.6, ω=0.200), with pairwise comparisons showing no differences across all groups.

#### Satisfaction With the Process of Information Delivery

**Figure 3:**
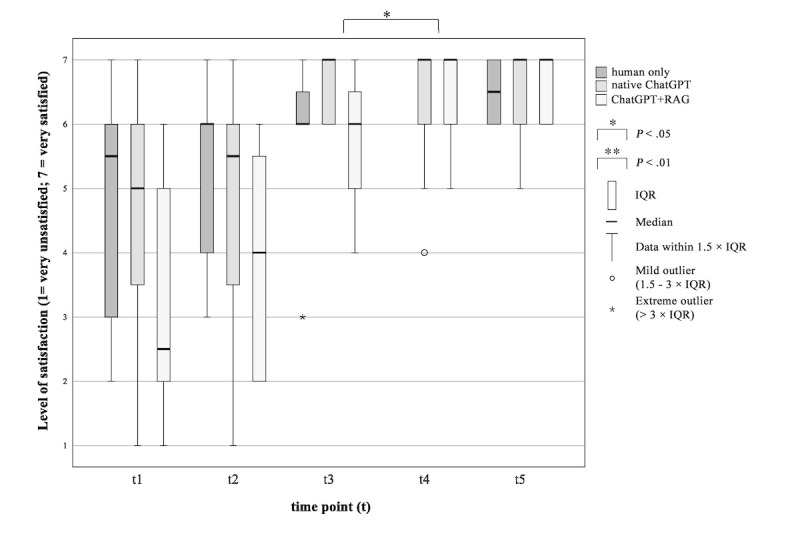
Patients’ satisfaction with the process of information delivery during the surgical informed consent consultation procedure. “How satisfied are you with the process of information delivery?” RAG: retrieval-augmented generation.

A difference in satisfaction levels was observed between the ChatGPT+RAG group and the native ChatGPT group (Figure 3). In the ChatGPT+RAG group, the patients were more satisfied with the process of information delivery after interacting with the AI chatbot (*P*=.014, *Z*=–2.460, *r*=0.710). Patients’ satisfaction with the process of information delivery in the native ChatGPT group does not differ after using an AI chatbot compared with after the physician explained the THA operation procedure (*P*=.28, *Z*=–1.089, *r*=0.314).

#### How Satisfied Are You With Your Perceived Level of Informedness?

**Figure 4:**
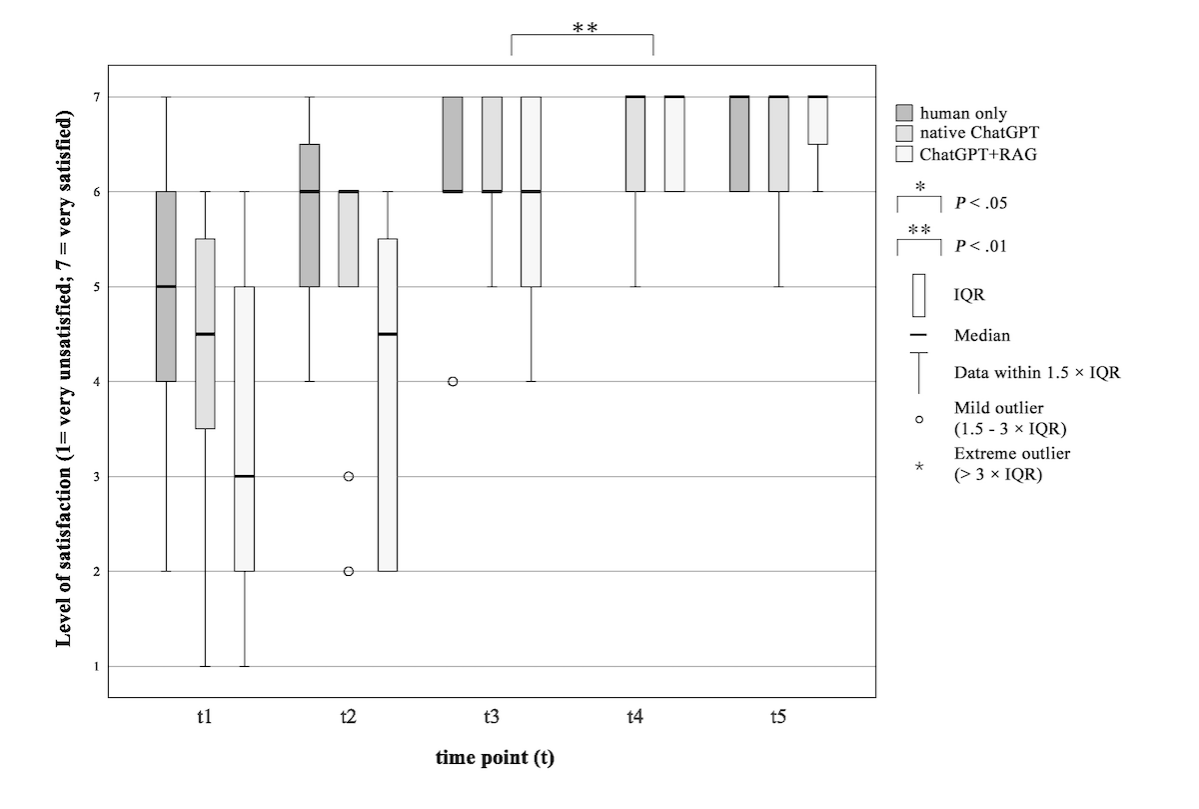
Patients’ satisfaction with the perceived level of informedness during the surgical informed consent consultation procedure. “How satisfied are you with your perceived level of informedness?” RAG: retrieval-augmented generation.

A difference in satisfaction levels was observed between the ChatGPT+RAG group and the native ChatGPT group (Figure 4). In the ChatGPT+RAG group, the patients were more satisfied with their level of informedness after interacting with the chatbot (*P*=.007, *Z*=–2.714, *r*=0.783). Patients’ satisfaction with the level of informedness in the native ChatGPT group does not differ after using the AI chatbot compared with after the physician explained the THA operation procedure (*P*=.06, *Z*=–1.890, *r*=0.546).

#### Satisfaction With the Amount of Information Received

**Figure 5:**
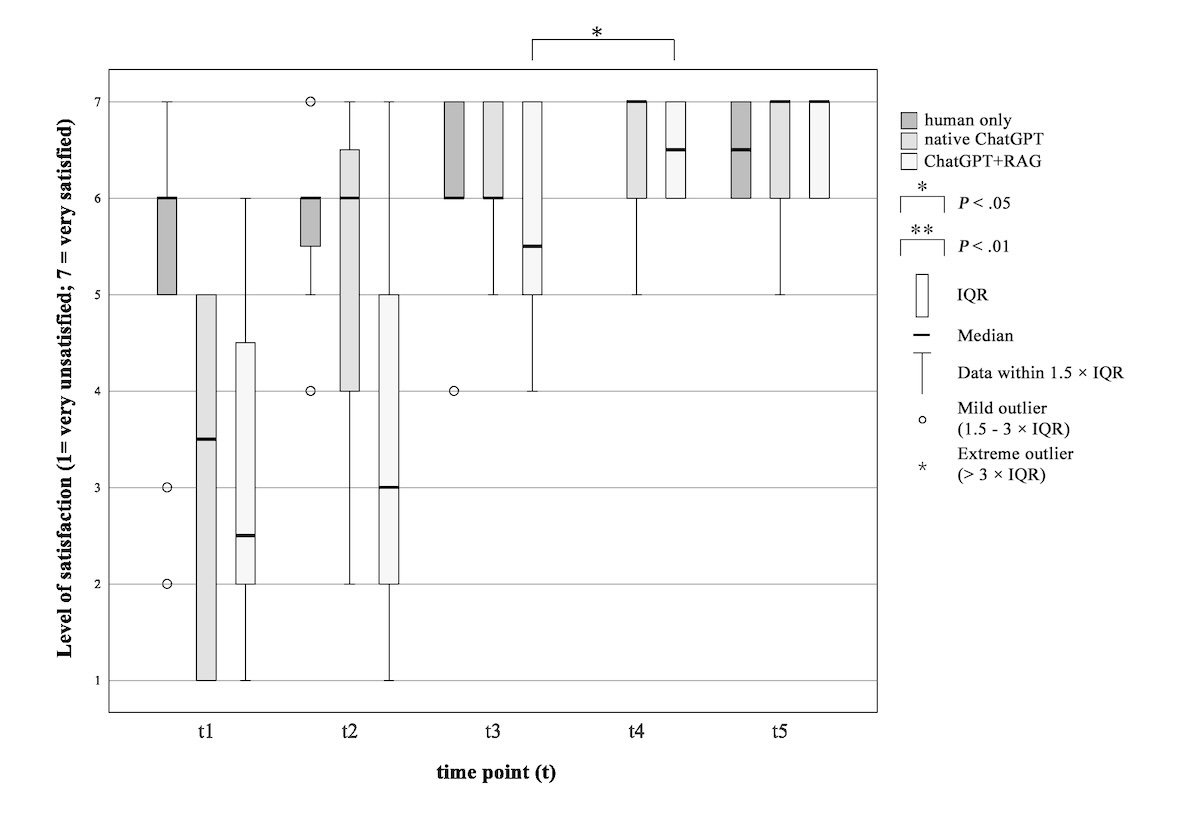
Patients’ satisfaction with the amount of information received during the surgical informed consent consultation procedure. “How satisfied are you with the amount of information received?” RAG: retrieval-augmented generation.

A difference in satisfaction levels with the quantity of information received was observed between the ChatGPT+RAG group and the native ChatGPT group (Figure 5). In the ChatGPT+RAG group, the patients were more satisfied with the level of information received after interacting with the AI chatbot (*P*=.02, *Z*=–2.310, *r*=0.667). Patients’ satisfaction levels in the native ChatGPT group do not differ after using the AI chatbot compared with after the physician explained the THA operation procedure (*P*=.32, *Z*=–1.000, *r*=0.289).

#### Preference for a Human Doctor Versus AI Chatbot

**Figure 6:**
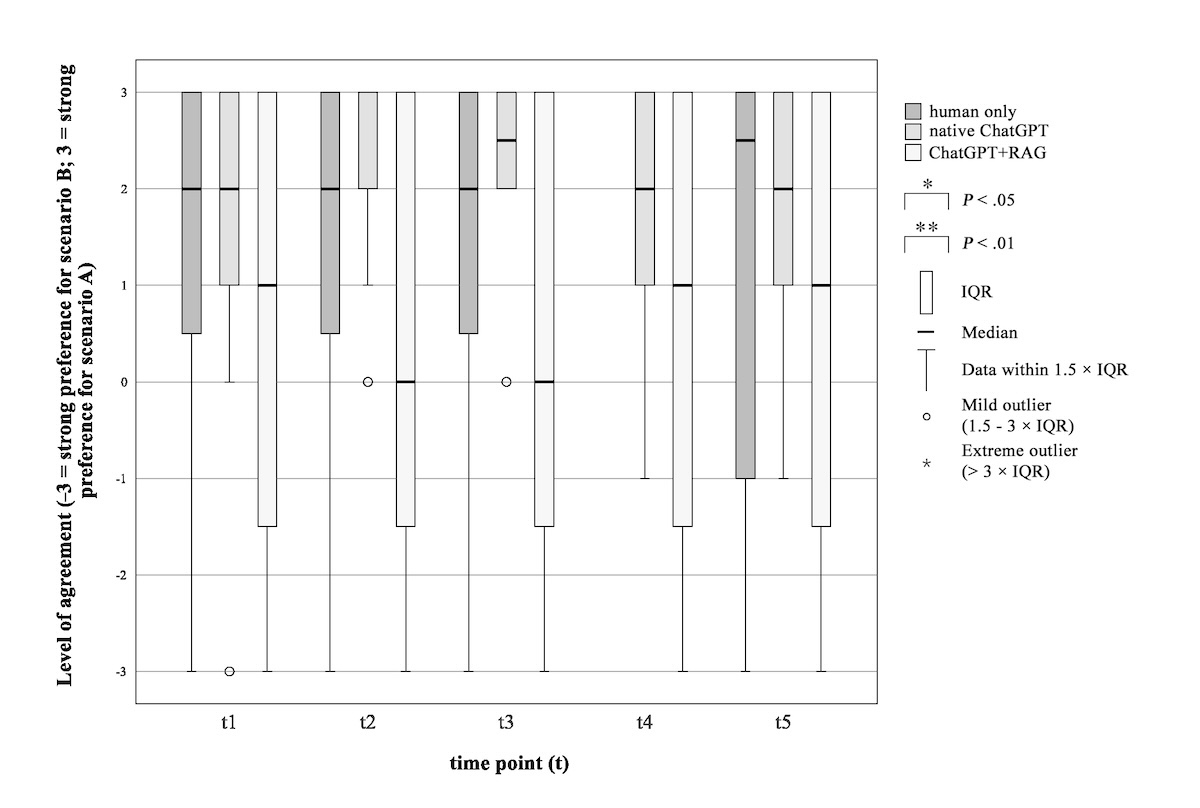
Patients’ preference for a human doctor versus an artificial intelligence chatbot during the surgical informed consent consultation procedure. “You have two different information scenarios, A and B. Which scenario do you prefer? Scenario A: Use of AI such as ChatGPT with significantly better quality of information, but without human physician contact. Scenario B: Information provided by a human physician, despite the information being of lower quality compared with the AI.” RAG: retrieval-augmented generation.

Results show that across all groups, participants preferred Scenario A (a human doctor with less accurate information) over Scenario B (an AI assistant alone with more accurate information). This preference was reflected in mean ratings ranging from 1.25 to 1.42 in the human-only group, 1.75 to 2.33 in the native ChatGPT group, and 0.58 to 0.83 in the ChatGPT+RAG group (Figure 6). The preferred scenario did not change during the procedure of informed consent (human only: *P*=.75, *χ*^2^_3_=1.200, ω=0.033; native ChatGPT: *P*=.78, *χ*^2^_4_=1.775, ωs=0.037; ChatGPT+RAG: *P*=.68, *χ*^2^_3_=1.500, ω=0.031).

### State-Trait Anxiety Inventory

The mean state anxiety among participants was 39.0% (SD 17.6%), with values ranging from 0% to 65.7%. The mean trait anxiety among participants was 28.9% (SD 14.4%), with values ranging from 0% to 61.4%. There was no correlation between State, Trait, and the preference of a physician in the informed consent procedure (p_state_=0.158; p_trait_=0.302), the level of satisfaction of informedness (p_state_=0.522; p_trait_=0.605), the procedure of information transfer (p_state_=0.856; p_trait_=0.822), the quantity of information (p_state_=0.566; p_trait_=0.330) as well as the preference for having a physician even with less information (p_state_=0.087; p_trait_=0.172).

### Sociodemographic Factors’ Impact on PPSQ Values

Regarding age, participants were divided into 2 groups (younger vs older; with 55% in the older group above the mean age of 63.16 years). A statistically significant difference was observed for satisfaction with the process of information delivery, with older participants reporting higher scores (U=99.00, *Z*=–1.991, *P*=.046, *r*=0.332). For all other items, no significant age-related differences were found: need for a human doctor in the informed consent consultation (U=140.00, *Z*=–0.703, *P*=.48), satisfaction level of informedness (U=133.50, *Z*=–0.864, *P*=.39), satisfaction with the amount of information (U=110.00, *Z*=–1.622, *P*=.11), and preference for a human doctor versus AI chatbot (U=147.50, *Z*=–0.411, *P*=.68).

For gender, a statistically significant difference was identified for satisfaction with the amount of information, with female participants reporting higher satisfaction than males (U=81.00, *Z*=–2.299, *P*=.02, *r*=0.383). No significant differences were found for need for a human doctor in the informed consent consultation (U=123.00, *Z*=–0.964, *P*=.34), satisfaction with the process of information delivery (U=98.50, *Z*=–1.722, *P*=.09), satisfaction level of informedness (U=100.50, *Z*=–1.652, *P*=.10), or preference for a human doctor versus AI chatbot (U=143.00, *Z*=–0.221, *P*=.83).

Analyses of educational level showed no statistically significant differences for any of the Likert items: need for a human doctor in the informed consent consultation (H=4.472, df=3, *P*=.22), satisfaction with the process of information delivery (H=2.538, df=3, *P*=.47), satisfaction level of informedness (H=1.018, df=3, *P*=.80), satisfaction with the amount of information (H=2.730, df=3, *P*=.44), and preference for a human doctor versus AI chatbot (H=5.823, df=3, *P*=.12).

### Health Literacy Impact on PPSQ Values

The HLT had a mean of 7.92 (SD 1.1) right answered questions, with a range from 6 to 9 right answers. No significant effects were identified related to the patients’ HLT outcomes. Analyses of HLT showed no statistically significant differences for any of the PPSQs: need for a human doctor in the informed consent consultation (H=2.435, df=3, *P*=.49), satisfaction with the process of information delivery (H=0.882, df=3, *P*=.83), satisfaction level of informedness (H=2.476, df=3, *P*=.48), satisfaction with the amount of information (H=2.919, df=3, *P*=.40), and preference for a human doctor versus AI chatbot (H=4.207, df=3, *P*=.24) with the patient’s highest level of education.

### The GAAIS

Patients’ attitude towards AI did not change after using the AI chatbot in the informed consent consultation, despite GAAIS being negative in the native ChatGPT group (*P*=.02, *Z*=–2251, *r*=0.650; Tables 2 and 3).

**Figure 7:**
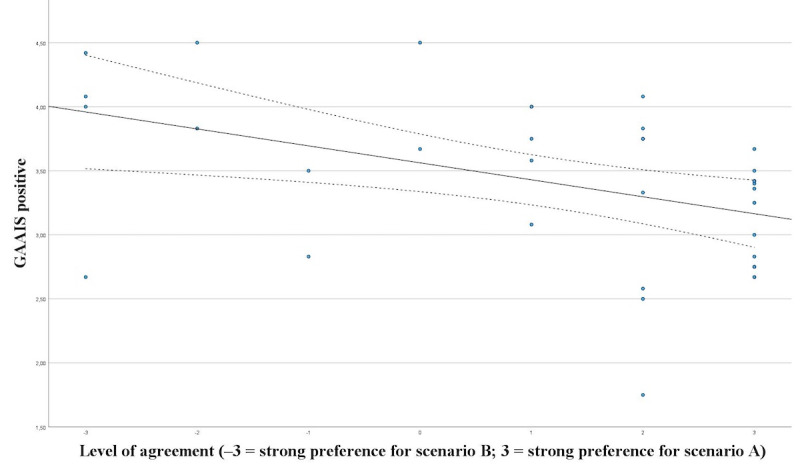
Correlation between GAAIS positive and preference of a physician in the informed consent procedure. GAAIS: General Attitudes towards Artificial Intelligence Scale.

**Figure 8:**
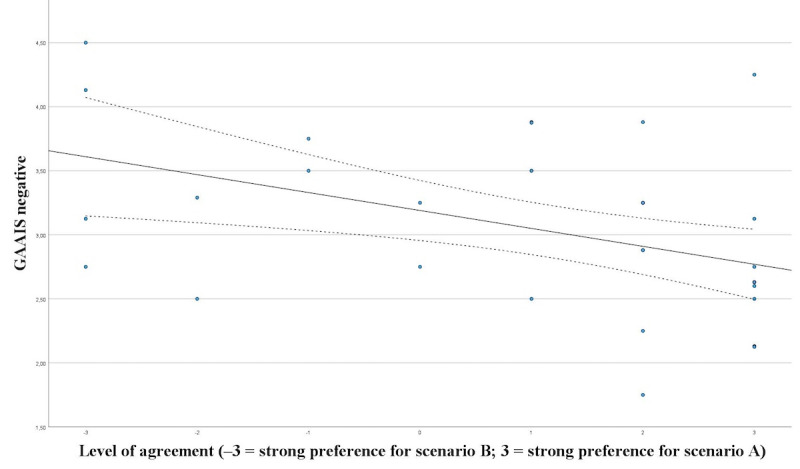
Correlation between GAAIS negative and preference of a physician in the informed consent procedure. GAAIS: General Attitudes towards Artificial Intelligence Scale.

Significant correlation was shown between GAAIS positive and preference of a physician in the informed consent procedure (*P*=.002; ρ=0.502; Figure 7) and between GAAIS negative and preference of a physician in the informed consent procedure (*P*=.002; ρ=0.500; Figure 8).

**Table 2:** Patients’ general attitude toward artificial intelligence before and after the surgical informed consent procedure.

GAAIS^a^	Preinformed consent (*t*=1)			Postinformed consent (*t*=5)		
	Human only	Native ChatGPT	ChatGPT+RAG^b^	Human only	Native ChatGPT	ChatGPT +RAG
Positive subscale, mean (SD)	3.2 (0.7)	3.6 (0.6)	3.5 (0.6)	3.1 (0.9)	3.6 (0.6)	3.4 (0.6)
Negative subscale, mean (SD)	2.7 (0.6)	3.2 (0.7)	3.2 (0.7)	2.7 (0.6)	3.4 (0.7)	3.3 (0.7)

^a^GAAIS: General Attitudes towards Artificial Intelligence Scale.

^b^RAG: retrieval-augmented generation.

**Table 3:** *P* values for patients’ general attitude toward artificial intelligence pre versus postsurgical informed consent procedure.

GAAIS^a^	Human only				Native ChatGPT				ChatGPT+RAG^b^		
	*P* value	Z	*r*	*P* value		Z	*r*	*P* value		Z	*r*
Pre versus post positive	.18	–1.334	0.385	*>.*99		0.000	0	.83		–0.210	0.061
Pre versus post negative	.87	–0.170	0.049	.02		–2251	0.650	.07		–1.841	0.531

^a^GAAIS: General Attitudes towards Artificial Intelligence Scale.

^b^RAG: retrieval-augmented generation.

### Patient Questions and AI Chatbot Responses

Examining the number of questions asked per group (Table 4), the control group stood out with fewer questions, totaling only 20, compared with the RAG group, which asked 52 questions (*P*=.002, *U*=20.50, *Z*=–3.042, *r*=0.621). The native ChatGPT group asked 39 questions, which were not statistically significant compared with the human-only group (*P*=.056, *U*=40.00, *Z*=–1.910, *r*=0.390). Additionally, no difference was observed between the RAG and native ChatGPT groups (*P*=0.22, *U*=51.00, *Z*=–1.228, *r*=0.251).

The average length of responses was measured for both intervention groups. In the native ChatGPT group, responses averaged 283.1 words and 2163.6 characters, while in the ChatGPT+RAG group, responses averaged 91.4 words and 689.8 characters (Table 5). This represents a ratio of 3.098 for word count (*P*<.001, *U*=81.00, *Z*=–7.483, *r*=0.784) and 3.136 for character count (*P*<.001, *U*=84.00, *Z*=–7.458, *r*=0.781), indicating that native ChatGPT-generated outputs are approximately 3 times longer than ChatGPT + RAG.

Table 6 shows that in both the native ChatGPT and ChatGPT+RAG groups, most patient questions were focused on the operation itself, followed by questions related to postoperative management. Fewer questions were asked about rehabilitation, daily life, and complications. No differences were observed between the 2 groups in the distribution of question classifications (*P*=.35, V=0.226).

**Table 4:** Number of questions asked by patients in each group.

	Human only (n=20)	Native ChatGPT (n=39)	ChatGPT+RAG^a^ (n=52)
Mean	1.67	3.25	4.33
Min, n	1	1	1
Max, n	4	7	7

^a^RAG: retrieval-augmented generation.

**Table 5:** Length of ChatGPT responses for both intervention groups.

	Native ChatGPT, mean (SD)	ChatGPT+RAG^a^, mean (SD)	Test for differences	
			*P* value	*r*
Words	283 (81.8)	91 (74.3)	<.001	0.784
Characters	2164 (463.2)	690 (558.1)	<.001	0.781

^a^RAG: retrieval-augmented generation.

**Table 6:** Percentage of patients’ questions per category in both intervention groups.

Category	Total (n=91), n (%)	Native ChatGPT (n=39), n (%)	ChatGPT+RAG^a^ (n=52), n (%)
Operation	39 (43)	16 (41)	23 (44)
Postoperative	21 (23)	7 (18)	14 (27)
Rehabilitation	11 (12)	4 (10)	7 (13)
Daily life	12 (13)	6 (15)	6 (12)
Complication	8 (9)	6 (15)	2 (4)

^a^RAG: retrieval-augmented generation.

### Evaluation of the ChatGPT Output Quality

Altogether, 91 questions were prompted, and the output was evaluated. Table 7 shows the results of physicians’ evaluation of the ChatGPT outputs for every category (relevance, accuracy, clarity, completeness, evidence-based, and the length of the output). ChatGPT+RAG had better outcomes in every category compared with native ChatGPT (*P*<.001; relevance: *r*=0.504, *U*=2110.00, *Z*=–6.803; accuracy: *r*=0.365, *U*=2503.50, *Z*=–4.920; clarity: *r*=0.565, *U*=1722.00, *Z*=–7.629; completeness: *r*=0.479, *U*=1977.50, *Z*=–6.463; evidence-based: *r*=0.483, *U*=2082.00, *Z*=–6.517; length: *r*=0.726, *U*=1168.00, *Z*=–9.795). The minimum value for ChatGPT+RAG was 4.625, while its maximum value was 4.961. The minimum value for native ChatGPT was 3.346, while its maximum value was 4.115. Across all outputs, the κ is 0.761 (SE=0.032, z=22.705, *P*<.001), indicating a substantial level of agreement.

We observed significantly fewer hallucinations in the RAG group versus in the native ChatGPT group (5 out of 52 [10%] vs 15 out of 39, 38%; Table 8). For 4 out of the 5 hallucinated responses in the ChatGPT+RAG group, no information was available in the FAQ documentation provided to ChatGPT via RAG from which the AI chatbot could draw.

ChatGPT+RAG has a significantly lower hallucination score compared with native ChatGPT (*P*=.002, *U*=729.00, *Z*=–3.168, *r*=0.332). The degree of severity shows no significant difference (*P*=.17, *U*=21.50, *Z*=–1.521, *r*=0.340).

Textboxes 1-3 show examples of mild, moderate, and alarming hallucinations identified in our study.

**Table 7:** Surgeons’ evaluation of ChatGPT output in both intervention groups.

	Native ChatGPT, mean (SD)	ChatGPT+RAG^a^, mean (SD)	Test for differences	
			*P* value	*r*
Relevance	4.115 (0.882)	4.856 (0.450)	<.001	0.504
Accuracy	3.910 (1.059)	4.625 (0.626)	<.001	0.365
Clarity	3.795 (1.036)	4.808 (0.484)	<.001	0.565
Completeness	3.667 (1.147)	4.644 (0.590)	<.001	0.479
Evidence-based	3.346 (1.356)	4.961 (0.583)	<.001	0.483
Length	3.346 (1.394)	4.962 (0.193)	<.001	0.726

^a^RAG: retrieval-augmented generation.

**Table 8:** Hallucinations in ChatGPT responses.

			Total		Native ChatGPT		ChatGPT+RAG^a^
Questions asked, n (%)			91 (100)		39 (100)		52 (100)
**Hallucinations, n (%)**							
	Not hallucinated	71 (78)		24 (62)		47 (90)	
	Hallucinated	20 (22)		15 (38)		5 (10)	
**Thereof, n (%)**							
	Partly hallucinated	15 (75)		12 (80)		3 (60)	
	Fully hallucinated	5 (25)		3 (20)		2 (40)	
**Level of severity, n (%)**							
	Mild severity	8 (40)		7 (47)		1 (20)	
	Moderate severity	9 (45)		7 (47)		2 (40)	
	Alarming severity	3 (15)		1 (6)		2 (40)	

^a^RAG: retrieval-augmented generation.

**Example of mild hallucination.**

- Patient question: “I am having a THA [total hip arthroplasty], how can I sleep after the operation?”
- Excerpt from the answer: “1. supine position: This is the preferred sleeping position immediately after the operation. Place a pillow between your legs to keep the hip in a stable position and prevent the operated leg from rotating inwards or outwards…”
- Explanation for categorization: Due to today’s prevailing minimally invasive surgical techniques, a cushion between the legs in supine position is no longer common or necessary.

**Example of moderate hallucination.**

- Patient question: “Which type of THA [total hip arthroplasty] does squeak?”
- Excerpt from the answer: “…ceramic-on-polyethylene bearing: This combination may also occasionally squeak, although this is less common than with pure ceramic-on-ceramic dentures...”
- Explanation for categorization: Under normal conditions, this bearing pair should not squeak. If this pair of bearings squeaks, the reason for the squeaking must be analyzed.

**Example of alarming hallucination.**

- Patient question: What do you do with a dislocated hip?
- Excerpt from the answer: “After reduction, an X-ray or MRI [Magnetic Resonance Imaging] is performed to ensure that the hip is positioned correctly and to rule out any associated injuries such as fractures or damage to ligaments and soft tissue…”
- Explanation for categorization: An X-ray must be performed before reduction to rule out fracture. No MRI is performed after the reduction, but a CT [Computed Tomography] scan may be performed in addition to the X-ray after reduction.

## Discussion

### Overview

The results of this study provide several insights into the potential use of AI chatbots like ChatGPT in health care, particularly in enhancing patient education during the surgical informed consent consultation procedure.

### Answers to Research Questions

#### What Are Patients’ Preferences Regarding the Use of an AI Chatbot in Conjunction With a Physician in Surgical Informed Consent in Comparison to a Physician Only?

Study results showed a clear expressed need for a human doctor in the informed consent procedure. No shifts in patients’ expressed needs were observed during the study procedure. Results also show a preference for scenario A (a human doctor only with worse information-delivery) versus scenario B (an AI-assistant only, with better information-delivery). The preferred scenario (ie, scenario A) did not change during the procedure of informed consent. However, the preference strength in each group differed at *t* value of 1 from 0.83 in the ChatGPT+RAG group to 1.83 in the native ChatGPT group with 1 being a slight preference for scenario A and 2 being a moderate preference for scenario A. The data presented suggest that patients would prefer the presence of a physician during the informed consent procedure. However, their preference for a human-only scenario (scenario A) over an AI chatbot-only scenario (scenario B) was only slight to moderate. This finding proposes the potential and additional benefit of patients’ interaction with AI chatbots.

In our study, state and trait anxiety exhibited no explanatory power for patients’ attitudes toward the necessity of a human doctor in the informed consent consultation, as well as their preference for a human doctor over an AI chatbot. In contrast, the general attitude towards AI (GAAIS) showed significant correlation with their preference toward interaction with AI during informed consent. One would assume that patients experiencing higher levels of anxiety may perceive AI-driven informed consent as less reliable compared with direct human interaction, potentially due to a need for empathetic communication. While it may be due to a lack of power with 36 patients, further research is necessary.

Li et al [38] explored the concerns surrounding the integration of AI into health care from both patient and physician perspectives. Key concerns are “algorithm aversion,” robophobia, loss of humanistic care, and interaction challenges. Patients are particularly concerned about trust, privacy, and AI’s ability to deal with rare conditions or individual circumstances. Physicians, on the other hand, are concerned about job security, legal liability, and the emotional toll of introducing AI technologies into their work environment. The authors emphasize the importance of fostering trust, improving AI-human interaction, and providing appropriate education and policy frameworks to mitigate these concerns.

Emotional states of patients, such as anxiety toward AI, should be specifically addressed when implementing AI systems in sensitive areas like informed consent.

Providing additional support, such as hybrid models combining AI with physician oversight, could help alleviate patient concerns and improve acceptance, particularly in patients with higher levels of anxiety.

#### How Does Patient Satisfaction Compare Across Informed Consent Procedures Involving Only a Physician, a Physician and ChatGPT, or a Physician and RAG-Integrated ChatGPT?

After the Q&A sessions, all groups are similarly satisfied with their respective level of informedness and the quantity of information they received. This means that the Q&A with the AI chatbot was able to satisfy the information needs of the patients similarly well compared with the physician answering questions. In the ChatGPT+RAG group, the AI-assistant was able to close the previously existing gaps in both the level of informedness and the quantity of information they received after the FAQ session.

In the ChatGPT+RAG group, the patients were more satisfied with the process of information delivery after the Q&A session compared with the human-only and native ChatGPT groups, which may be due to the quality of responses observed with that approach (discussed below). The physicians involved in the study also reported that the more concise responses in the ChatGPT+RAG group were seen as very positive by the patients.

The improved satisfaction in the RAG group may be attributed to the more context-specific and evidence-based responses generated by the RAG-enhanced system. These findings are consistent with prior research indicating that the RAG approach improves output quality, increases patient confidence, and understanding during medical consultations in patient-AI chatbot interaction [39-41]. By tailoring responses to individual questions and incorporating subject- and clinic-specific data, the RAG approach appears to address key patient concerns more effectively than the native ChatGPT model.

One of the most striking findings was that the control group, which did not interact with the AI chatbot, asked the fewest questions (n=20), whereas the ChatGPT group asked more (n=39), and the RAG group asked the most questions (n=52). The higher number of questions observed in the AI-supported groups may reflect differences in interaction dynamics rather than a lack of clarity, relevance, quality, or completeness in the responses, which were all rated as good to very good, particularly in the ChatGPT+RAG group (Table 7). Patients may have felt more comfortable, autonomous, or curious when interacting with the AI chatbot, especially in the ChatGPT+RAG group, where responses tended to be more concise and focused. This may have encouraged additional follow-up questions and deeper engagement with the content. A general indication of these results is therefore that patients in informed consent procedures can benefit from multimedia decision aids and tools such as ChatGPT that may help to encourage engagement and improve patient understanding [42].

#### How Does the Integration of RAG With ChatGPT Improve the Quality of Responses to Patients’ Questions Compared With the Native ChatGPT Model?

The results clearly demonstrate the advantages of the RAG approach over native ChatGPT in all measured quality dimensions. Responses generated with ChatGPT+RAG consistently scored higher in relevance, accuracy, clarity, completeness, evidence-based, and length compared with those generated by the native ChatGPT model (Table 7). This demonstrates the effectiveness of incorporating domain-specific information into AI chatbot responses to ensure better alignment with patient needs.

ChatGPT+RAG responses were significantly more concise than native ChatGPT responses, which tended to be much longer and more detailed. While detailed responses may seem advantageous, overly long responses run the risk of overwhelming patients and diluting the focus on their specific questions. In contrast, the concise responses of ChatGPT+RAG were likely perceived as clearer and more directly relevant, contributing to the higher quality ratings observed.

The use of RAG significantly reduced hallucinations in our study. In 4 out of 5 cases in which the AI chatbot hallucinated in the ChatGPT+RAG group, hallucinations were due to missing information in the FAQ document provided as information to the AI chatbot. This might suggest that when properly designed and managed, AI chatbots can provide responses with very few hallucinations. Other studies confirmed the effectiveness of a RAG approach für medical AI chatbots [39-41,43,44]. The low error rate obtainable through RAG in conjunction with as complete as possible and guideline-compliant FAQ documentation, together with the high consistency of an AI chatbot, can therefore lead to very good results in combination with a human in medical education. Furthermore, the minimal hallucinations observed in the ChatGPT+RAG group provided valuable insights, enabling improvements to our internal FAQ documentation following the study.

While our study did not explicitly measure consistency across identical or similar prompts, we observed qualitative indications that ChatGPT combined with RAG exhibited higher consistency in its responses. For instance, in the native ChatGPT condition, answers to similar questions regarding the expected length of hospital stay varied, once stating “3-7 days” and another time “5-10 days.” In contrast, the RAG-enhanced version consistently responded with “4-7 days,” which aligns precisely with the information provided in the underlying FAQ documentation. This suggests that the integration of a structured reference source via RAG can improve both accuracy and consistency. However, due to the limited number of participants and the small number of semantically similar questions per group, we were not able to systematically evaluate consistency in this study.

The results show the value of the RAG approach optimizing AI chatbots for use case-specific and clinic-specific information, particularly in sensitive processes like surgical informed consent consultations, where correct, clear, and comprehensive communication is essential. However, it is important to continuously monitor these tools to ensure patient safety in all cases. Furthermore, more research is needed with different datasets provided to the AI chatbot via the RAG approach, different AI-language models, different RAG-architectures, different prompts, and with more patients.

#### How do Patient Sociodemographic Characteristics and General Attitude Toward AI Influence Patients’ Preference Toward Using an AI Chatbot in Surgical Informed Consent?

Our results show that age, gender, and education did not influence patients’ preference toward using an AI chatbot in surgical informed consent. There is no clear consensus as per today in the body of literature about the role of age in AI or technology acceptance. Witkowski et al [45] found that age is associated with AI discomfort in health care. Miller et al [46], who studied patients’ use and perception of an AI-based symptom assessment and advice technology in a British primary care waiting room, identified several age-related trends among respondents, with a directional trend for more young respondents (18- to 24-year-olds) to report that the tool provided helpful advice than older respondents (adults older than 70 years). Other studies show that older people are not per se “anti-tech” or “anti-AI.” Xiang et al [47] found that receptivity to medical AI increases with age, reaching its highest levels among individuals aged 49 years and older. More research is required in different age groups and medical domains, for example, in arthroscopy, where patients are usually younger.

Patients’ general attitudes toward AI demonstrated weak explanatory power for patients’ preference regarding the necessity of a doctor in the informed consent process and their preference for a human doctor over an AI chatbot. However, further research is needed to better understand the impact of attitudes toward AI. The general attitude of patients toward AI could be considered in the design of processes and systems in the future. This is also against the background that AI and systems that integrate AI are developing rapidly, and their implementation in interaction with patients and doctors must therefore be continuously examined.

The results of the present study demonstrate that, although the analysis of native ChatGPT outputs shows significantly worse performance compared with ChatGPT+RAG, patients report high satisfaction with the process of information delivery, their perceived level of informedness, and the amount of information received across all 3 groups. This should be considered in the design of AI-driven patient interactions. Despite the suboptimal quality of native ChatGPT responses as evaluated by physicians, the RAG approach showed significantly better performance, including higher patient engagement and markedly fewer hallucinations, making it a more promising option for precise patient information delivery.

However, given that patients prefer the presence of a physician and that the RAG approach cannot eliminate hallucinations, we strongly recommend using RAG-based AI chatbots, but only in combination with a physician. Ensuring that patients are not left alone in the information process is crucial. RAG chatbots, meanwhile, offer a complementary solution for enhancing patient communication and reducing the time burden on physicians in daily practice. The additional time gained can help address issues related to patient education, literacy barriers, language differences, and limited doctor communication skills (eg, offering an easy-language mode).

### Future Research Direction

The findings of this study suggest several potential directions for future research to better understand patient preferences and concerns regarding AI chatbots in health care, as well as to explore their possible applications in patient care. As we observed more questions in the AI-assisted groups, these tools may help enhance patient engagement. However, the underlying interaction dynamics as well as the number, type, and nature of follow-up questions should be explored in future research.

Since the RAG approach has proven to have a significantly higher engagement and quality compared with the native ChatGPT version, it might be advisable to prioritize further investigation and to expand the approach by incorporating patient-specific input. Further investigation is needed to assess AI chatbots that could provide patient-tailored information and guidance during preoperative and postoperative care.

From a technological perspective, further work is needed to examine how different implementations, such as variations in the RAG framework or AI-language models, and variations in the type and structure of information provided to and by the AI chatbot, affect the results. Future studies should also explore patient interactions using different interfaces (eg, keyboards or voice assistants) and mobile apps of AI chatbots, particularly outside formal informed consent procedures.

The potential for these tools to be perceived as reliable and trustworthy by patients requires careful evaluation while adhering to the principles of patient-centered care [45]. Patients may also have concerns regarding data privacy and the ability of AI systems to handle rare or complex conditions. While this pilot study did not explore these dimensions in depth, such concerns are central to the responsible use of AI in health care and should be addressed in future research. Future research should integrate qualitative methods, such as interviews or free-text survey responses, to complement structured evaluations and capture deeper insights into patient trust, expectations, and experiences with AI.

Finally, demographic factors such as age, cultural background, and other sociodemographic variables need to be studied in greater detail to understand how different patient groups perceive and interact with AI chatbots. While AI has the potential to make health care more accessible, it is essential to ensure that these tools address the diverse needs of patients and do not inadvertently create new barriers to care.

### Limitations

In our study, we aimed at covering a wide range of questions regarding the new field of patient AI chatbot interaction in the surgical informed consent procedure. Yet, our study is not without limitations.

First, the relatively small sample size (N=36) limits the statistical power and generalizability of the findings. However, this sample size was deliberately chosen, as the exploratory pilot study aimed at hypothesis generation and feasibility testing. Future research should expand the sample size to enable more robust subgroup analyses. Second, the over-representation of middle-aged and older adults in our sample may limit the applicability of findings to younger patient populations. While we addressed age-related aspects through subgroup analysis using commonly applied age categories in health care and HCI literature, future studies should aim to recruit more age-diverse cohorts to better understand age differences in trust, comprehension, and preferences regarding AI-assisted consent formats. Third, a physician was present during all informed consent consultations across all study groups. This ensured ethical oversight and patient safety, particularly in the AI-assisted scenarios, but may have influenced patient behavior and interaction with the chatbot. Patients might have perceived the chatbot as being supervised or endorsed by the physician. Future studies should explore different formats and levels of physician involvement to better isolate the effects of AI guidance.

To address these limitations and build on our findings, future studies should involve larger and more demographically diverse samples, test a range of physician-AI interaction models, and include qualitative measures. Moreover, replication in varied clinical settings will be essential to assess the generalizability and practical utility of AI chatbots in surgical informed consent consultations and other medical communication contexts.

### Conclusions

This study is the first to explore the feasibility, response quality, and patient acceptance of AI-supported surgical informed consent consultations. By comparing traditional physician-led consultations with those assisted by ChatGPT and ChatGPT+RAG, we found that patient satisfaction remained consistently high across all groups, suggesting that the use of AI support did not compromise the informed consent experience. The integration of RAG significantly enhanced the quality of AI-generated responses and reduced hallucination rates compared with native ChatGPT. Notably, participants in the ChatGPT+RAG group reported the highest satisfaction with information delivery and perceived informedness, alongside greater engagement as reflected in the number of questions asked.

Although most patients still preferred physician-led consultations, our findings are encouraging. They indicate that AI chatbots, particularly when enhanced with clinical-specific information via RAG, can deliver accurate, context-aware, and concise information while promoting patient participation. Given the small sample size, future studies are needed to confirm these findings in more diverse populations and to examine the role of AI chatbots in different and more autonomous patient-AI interactions and across different clinical contexts. However, as we embrace this opportunity, it is essential to remain cautious about challenges such as trust, data privacy, and the preservation of the human touch in patient care.

## Appendix

Multimedia Appendix 1 Questionnaires, German and English Version.

Multimedia Appendix 2 Hallucination examples.

## Abbreviations

AI: artificial intelligence
FAQ: frequently asked questions
GAAIS: General Attitudes towards Artificial Intelligence Scale
GPT: generative pretrained transformer
HLT: Health Literacy Test
PPSQ: Patients’ Preferences and Satisfaction Questionnaire
Q&A: questions and answers
RAG: retrieval-augmented generation
THA: total hip arthroplasty

## Data Availability

The datasets generated or analyzed during this study are not publicly available due to privacy concerns, but are available from the corresponding author on reasonable request. Sample dialogues are provided in the Multimedia Appendix 2.

## References

[1] Lee P, Bubeck S, Petro J (2023). Benefits, limits, and risks of GPT-4 as an AI Chatbot for medicine. N Engl J Med.

[2] Draschl A, Hauer G, Fischerauer SF, Kogler A, Leitner L, Andreou D, Leithner A, Sadoghi P (2023). Are ChatGPT's free-text responses on periprosthetic joint infections of the hip and knee reliable and useful?. J Clin Med.

[3] Li Y, Liang S, Zhu B, Liu X, Li J, Chen D, Qin J, Bressington D (2023). Feasibility and effectiveness of artificial intelligence-driven conversational agents in healthcare interventions: a systematic review of randomized controlled trials. Int J Nurs Stud.

[4] Gabarron E, Larbi D, Denecke K, Årsand E (2020). What do we know about the use of chatbots for public health?. Stud Health Technol Inform.

[5] Caldarini G, Jaf S, McGarry K (2022). A literature survey of recent advances in chatbots. Information.

[6] Shah A, Wahood S, Guermazi D, Brem CE, Saliba E (2024). Skin and syntax: large language models in dermatopathology. Dermatopathology (Basel).

[7] Ayers JW, Poliak A, Dredze M, Leas EC, Zhu Z, Kelley JB, Faix DJ, Goodman AM, Longhurst CA, Hogarth M, Smith DM (2023). Comparing physician and artificial intelligence chatbot responses to patient questions posted to a public social media forum. JAMA Intern Med.

[8] Suppadungsuk S, Thongprayoon C, Miao J, Krisanapan P, Qureshi F, Kashani K, Cheungpasitporn W (2023). Exploring the potential of chatbots in critical care nephrology. Medicines (Basel).

[9] Kienzle A, Niemann M, Meller S, Gwinner C (2024). ChatGPT may offer an adequate substitute for informed consent to patients prior to total knee arthroplasty-yet caution is needed. J Pers Med.

[10] Li J, Cheng X, Zhao X (2023). HaluEval: a large-scale hallucination evaluation benchmark for large language models.

[11] Achiam J, Adler S, OpenAI (2023). GPT-4 technical report. arXiv.

[12] Lee GU, Hong DY, Kim SY (2024). Comparison of the problem-solving performance of ChatGPT-3.5, ChatGPT-4, Bing Chat, and Bard for the Korean emergency medicine board examination question bank. Medicine.

[13] Xue E, Bracken-Clarke D, Iannantuono GM, Choo-Wosoba H, Gulley JL, Floudas CS (2024). Utility of large language models for health care professionals and patients in navigating hematopoietic stem cell transplantation: comparison of the performance of ChatGPT-3.5, ChatGPT-4, and Bard. J Med Internet Res.

[14] Alessandri-Bonetti M, Liu HY, Donovan JM, Ziembicki JA, Egro FM (2024). A comparative analysis of ChatGPT, ChatGPT-4, and Google Bard performances at the advanced burn life support exam. J Burn Care Res.

[15] Sabaner MC, Hashas ASK, Mutibayraktaroglu KM, Yozgat Z, Klefter ON, Subhi Y (2024). The performance of artificial intelligence-based large language models on ophthalmology-related questions in Swedish proficiency test for medicine: ChatGPT-4 omni vs Gemini 1.5 Pro. AJO Int.

[16] Shahriar S, Lund B, Mannuru NR (2024). Putting GPT-4o to the sword: a comprehensive evaluation of language, vision, speech, and multimodal proficiency. arXiv.

[17] Goodman RS, Patrinely JR, Osterman T, Wheless L, Johnson DB (2023). On the cusp: considering the impact of artificial intelligence language models in healthcare. Med.

[18] Deutsche Gesellschaft für Orthopädie und Unfallchirurgie e.V. (DGOU) (2021). Evidenz- und konsensbasierte Indikationskriterien zur Hüfttotalendoprothese bei Coxarthrose [evidence-based and consensus-based indication criteria for total hip replacement in coxarthrosis]. S3-Leitlinie [S3 guidlines].

[19] Wainwright TW, Gill M, McDonald DA (2020). Consensus statement for perioperative care in total hip replacement and total knee replacement surgery: Enhanced Recovery After Surgery (ERAS®) society recommendations. Acta Orthop.

[20] Zampogna B, Parisi FR, Zampoli A, Papalia R (2024). Artificial intelligence in orthopaedic surgery made easy. Springer.

[21] Learmonth ID, Young C, Rorabeck C (2007). The operation of the century: total hip replacement. Lancet.

[22] Trousdale RT, McGrory BJ, Berry DJ, Becker MW, Harmsen WS (1999). Patients' concerns prior to undergoing total hip and total knee arthroplasty. Mayo Clin Proc.

[23] Macario A, Schilling P, Rubio R, Bhalla A, Goodman S (2003). What questions do patients undergoing lower extremity joint replacement surgery have?. BMC Health Serv Res.

[24] Marinelli V, Danzi OP, Mazzi MA, Secchettin E, Tuveri M, Bonamini D, Rimondini M, Salvia R, Bassi C, Del Piccolo L (2020). PREPARE: PreoPerative anxiety REduction. one-year feasibility RCT on a brief psychological intervention for pancreatic cancer patients prior to major surgery. Front Psychol.

[25] Chiu PL, Li H, Yap KY, Lam KMC, Yip PLR, Wong CL (2023). Virtual reality-based intervention to reduce preoperative anxiety in adults undergoing elective surgery: a randomized clinical trial. JAMA Netw Open.

[26] Sepucha KR, Atlas SJ, Chang Y, Freiberg A, Malchau H, Mangla M, Rubash H, Simmons LH, Cha T (2018). Informed, patient-centered decisions associated with better health outcomes in orthopedics: prospective cohort study. Med Decis Making.

[27] Narayanan AS, Stoll KE, Pratson LF, Lin FC, Olcott CW, Del Gaizo DJ (2021). Musculoskeletal health literacy is associated with outcome and satisfaction of total knee arthroplasty. J Arthroplasty.

[28] Schepman A, Rodway P (2020). Initial validation of the general attitudes towards artificial intelligence scale. Comput Hum Behav Rep.

[29] CD S, RL G, Lushene R (1970). The manual for the State-Trait Anxiety Inventory.

[30] Grimm J (2009). State-trait-anxiety inventory nach Spielberger [state-trait anxiety inventory based on Spielberger]. Deutsche Lang- und Kurzversion [German long and short versions]. MF-Working Paper.

[31] Magruder ML, Rodriguez AN, Wong JCJ, Erez O, Piuzzi NS, Scuderi GR, Slover JD, Oh JH, Schwarzkopf R, Chen AF, Iorio R, Goodman SB, Mont MA (2024). Assessing ability for ChatGPT to answer total knee arthroplasty-related questions. J Arthroplasty.

[32] AE - Deutsche Gesellschaft für Endoprothetik e.V. Häufig gestellte Fragen zur Hüfte [frequently asked questions about the hip]. AE - Deutsche Gesellschaft für Endoprothetik e.V. [AE - German Society for Endoprosthetics e.V.].

[33] Huang L, Yu W, Ma W, Zhong W, Feng Z, Wang H, Chen Q, Peng W, Feng X, Qin B, Liu T (2025). A survey on hallucination in large language models: principles, taxonomy, challenges, and open questions. ACM Trans Inf Syst.

[34] Maynez J, Narayan S, Bohnet B, McDonald R (2020). On faithfulness and factuality in abstractive summarization. Proc 58th Annu Meet Assoc Comput Linguistics.

[35] Rawte V, Chakraborty S, Pathak A (2023). The troubling emergence of hallucination in large language models - an extensive definition, quantification, and prescriptive remediations. arXiv.

[36] Julious SA (2005). Sample size of 12 per group rule of thumb for a pilot study. Pharm Stat.

[37] Landis JR, Koch GG (1977). The measurement of observer agreement for categorical data. Biometrics.

[38] Li W, Liu X (2025). Anxiety about artificial intelligence from patient and doctor-physician. Patient Educ Couns.

[39] Sanna L, Bellan P, Magnolini S (2024). Building certified medical chatbots: overcoming unstructured data limitations with modular RAG.

[40] Li A, Shrestha R, Jegatheeswaran T, Chan HO, Hong C, Joshi R (2024). Mitigating hallucinations in large language models: a comparative study of RAG-enhanced vs. human-generated medical templates. MedRxiv.

[41] Kirubakaran S, Kathrine J, Kanaga E (2024). A RAG-based medical assistant especially for infectious diseases.

[42] Porter AL, Ebot J, Lane K, Mooney LH, Lannen AM, Richie EM, Dlugash R, Mayo S, Brott TG, Ziai W, Freeman WD, Hanley DF (2020). Enhancing the informed consent process using shared decision making and consent refusal data from the CLEAR III trial. Neurocrit Care.

[43] Lewis P, Perez E, Piktus A, Petroni F, Karpukhin V, Goyal N (2020). Retrieval-augmented generation for knowledge-intensive NLP tasks. arXiv.

[44] Martino A, Iannelli M, Truong C (2023). The semantic web: ESWC 2023 satellite events, hersonissos, crete, greece. Lect Notes Comput Sci.

[45] Witkowski K, Dougherty RB, Neely SR (2024). Public perceptions of artificial intelligence in healthcare: ethical concerns and opportunities for patient-centered care. BMC Med Ethics.

[46] Miller S, Gilbert S, Virani V, Wicks P (2020). Patients' utilization and perception of an artificial intelligence-based symptom assessment and advice technology in a British primary care waiting room: exploratory pilot study. JMIR Hum Factors.

[47] Xiang Y, Zhao L, Liu Z, Wu X, Chen J, Long E, Lin D, Zhu Y, Chen C, Lin Z, Lin H (2020). Implementation of artificial intelligence in medicine: status analysis and development suggestions. Artif Intell Med.

