# Supplementary material for: Exploring Patient Perspectives, Engagement, and Output Quality in Doctor-Supervised Use of Artificial Intelligence During Informed Consent Consultation With ChatGPT and Retrieval Augmented Generation (RAG): Quantitative Exploratory Study
Source: J Med Internet Res. 2025 Oct 22;27:e73717. doi: 10.2196/73717 (PMC12590045; doi:10.2196/73717)
Supplement: Multimedia Appendix 1 [file jmir_v27i1e73717_app1.docx]

# Appendix 1: Questionnaires, German and English Version

## Patients’ preferences and satisfaction questionnaire (PPSQ)

| PPSQ1 |  |
| --- | --- |
| Wie zufrieden sind Sie mit folgender Aussage: Ein/e menschliche Ärzt:in ist für den Prozess der chirurgischen Einwilligung notwendig. | "To what extent do you agree with the following statement: The involvement of a human doctor is essential in the surgical consent process." |
|  |  |
| PPSQ 2 |  |
| Wie zufrieden sind Sie mit dem Prozess der Informationsvermittlung? | How satisfied are you with the process of information delivery? |
|  |  |
| PPSQ 3 |  |
| Wie zufrieden sind Sie mit Ihrem Informationsstand? | How satisfied are you with your perceived level of informedness? |
|  |  |
| PPSQ 4 |  |
| Wie zufrieden sind Sie mit dem Umfang der erhaltenen Informationen? | How satisfied are you with the amount of information received? |
|  |  |
| PPSQ 5 |  |
| Sie haben zwei unterschiedlichen Aufklärungsszenarien A und B. Setzen Sie ein Kreuz bei Ihrer Präferenz.  Szenario A: Verwendung von KI wie ChatGPT mit wesentlich besserer Informationsausgabe, aber ohne menschlichen ärztlichen Kontakt.  Szenario B: Die Aufklärung durch ein/e menschliche Ärzt:in trotz einer schlechteren Informationsausgabe als durch die KI. | You have two different information scenarios, A and B. Please mark your preference with an X.  Scenario A: Use of AI such as ChatGPT with significantly better quality of information, but without human physician contact.  Scenario B: Information provided by a human physician, despite the information being of lower quality compared to the AI. |

## Health Literacy Test (HLT) for Total Hip Arthroplasty

### **German version**

1. Was versteht man unter Hüftarthrose?
   1. Eine degenerative Gelenkerkrankung, die Entzündungen und Schmerzen im Hüftgelenk verursacht
   2. Eine genetisch bedingte Störung, die die Knochen und Muskeln im Hüftgelenk betrifft
   3. Eine Virusinfektion, die zu Schwellungen und Steifheit in der Hüfte führt
   4. Ein Zustand, der durch übermäßige körperliche Aktivität verursacht wird und das Hüftgelenk schädigt
2. Was ist eine Hüftgelenkstotalendoprothese?
   1. Ein chirurgischer Eingriff, bei dem das gesamte Hüftgelenk entfernt und durch ein künstliches Gelenk ersetzt wird
   2. Eine nicht-chirurgische Behandlung, bei der die Symptome der Hüftarthrose mit Medikamenten behandelt werden
   3. Ein Übungsprogramm zur Stärkung der Muskeln um das Hüftgelenk
   4. Eine physikalische Therapietechnik, bei der manuelle Manipulationen zur Verbesserung der Hüftbeweglichkeit eingesetzt werden
3. Was sind die häufigsten Symptome einer Hüftarthrose?
   1. Gelenkschmerzen, Steifheit, Schwellungen und Schwierigkeiten beim Gehen oder Bewegen der Hüfte
   2. Fieber, Schüttelfrost und Schwäche im Hüftgelenk
   3. Muskelkrämpfe und Spasmen im Bein und der Hüfte
   4. Kribbeln und Taubheitsgefühl in der Hüftgegend
4. Wie lange ist die typische Genesungszeit nach einer Hüft-Totalendoprothese?
   1. 1-2 Wochen
   2. 4-6 Wochen
   3. 3-6 Monate
   4. 1 Jahr oder länger
5. Welche Risikofaktoren können das Risiko einer postoperativen Infektion nach einer Hüft-Totalendoprothese erhöhen?
   1. Rauchen
   2. Adipositas
   3. Diabetes
   4. Alle der oben genannten Punkte
6. Können Sie kurz die Anatomie des Hüftgelenks beschreiben?
   1. Das Hüftgelenk besteht aus zwei Hauptknochen: dem Azetabulum und dem Femur.
   2. Das Hüftgelenk besteht aus drei Hauptknochen: dem Oberarmknochen, der Elle und der Speiche.
   3. Das Hüftgelenk besteht aus drei Hauptknochen: dem Oberschenkelknochen, der Kniescheibe und dem Schienbein.
   4. Das Hüftgelenk besteht aus drei Hauptknochen: dem Oberarmknochen, der Speiche und dem Wadenbein.
7. Welche der folgenden Komplikationen kann nach einer Hüft-Totalendoprothese auftreten?
   1. Blutgerinnsel
   2. Zahnfleischerkrankung
   3. Augeninfektionen
   4. Ohrinfektionen
8. Welches ist ein spezifisches Risiko, das nach einer Hüft-Totalendoprothesen-Operation auftreten kann?
   1. Schlaflosigkeit
   2. Hautausschläge
   3. Schädigung der Nerven
   4. Haarausfall
9. Welche der folgenden Verhaltensweisen können die Genesung nach einer Hüft-Totalendoprothese positiv beeinflussen?
   1. Längere sitzende Tätigkeit und Vermeidung von Bewegung
   2. Regelmäßige Physiotherapie und Bewegung entsprechend den Empfehlungen der Ärzt:Innen
   3. Ignorieren von Anzeichen einer Infektion oder von Komplikationen
   4. Verzehr von zuckerhaltigen Lebensmitteln und Getränken

### **English version**

1. What is hip osteoarthritis?
   1. A degenerative joint disease that causes inflammation and pain in the hip joint
   2. A genetic disorder that affects the bones and muscles in the hip joint
   3. A viral infection that leads to swelling and stiffness in the hip
   4. A condition caused by excessive physical activity that damages the hip joint
2. What is a total hip replacement?
   1. A surgical procedure in which the entire hip joint is removed and replaced with an artificial joint
   2. A non-surgical treatment in which the symptoms of hip osteoarthritis are treated with medication
   3. An exercise program to strengthen the muscles around the hip joint
   4. A physical therapy technique involving manual manipulations to improve hip mobility
3. What are the most common symptoms of hip osteoarthritis?
   1. Joint pain, stiffness, swelling, and difficulty walking or moving the hip
   2. Fever, chills, and weakness in the hip joint
   3. Muscle cramps and spasms in the leg and hip
   4. Tingling and numbness in the hip area
4. What is the typical recovery time after a total hip replacement?
   1. 1–2 weeks
   2. 4–6 weeks
   3. 3–6 months
   4. 1 year or longer
5. Which risk factors can increase the likelihood of a postoperative infection after a total hip replacement?
   1. Smoking
   2. Obesity
   3. Diabetes
   4. All of the above
6. Can you briefly describe the anatomy of the hip joint?
   1. The hip joint consists of two main bones: the acetabulum and the femur
   2. The hip joint consists of three main bones: the humerus, ulna, and radius
   3. The hip joint consists of three main bones: the femur, patella, and tibia
   4. The hip joint consists of three main bones: the humerus, radius, and fibula
7. Which of the following complications can occur after a total hip replacement?
   1. Blood clots
   2. Gum disease
   3. Eye infections
   4. Ear infections
8. What is a specific risk that can occur after a total hip replacement surgery?
   1. Insomnia
   2. Skin rashes
   3. Nerve damage
   4. Hair loss
9. Which of the following behaviors can positively influence recovery after a total hip replacement?
   1. Prolonged sitting and avoiding movement
   2. Regular physiotherapy and exercise according to doctors’ recommendations
   3. Ignoring signs of infection or complications
   4. Consuming sugary foods and drinks

## The General Attitudes towards Artificial Intelligence Scale (GAAIS)

**English version**

| Subscale (not for display) | Number (not for display) | Item |
| --- | --- | --- |
| Positive | 1 | For routine transactions, I would rather interact with an artificially intelligent system than with a human. |
| Positive | 2 | Artificial Intelligence can provide new economic opportunities for this country. |
| Negative | 3 | Organisations use Artificial Intelligence unethically. |
| Positive | 4 | Artificially intelligent systems can help people feel happier. |
| Positive | 5 | I am impressed by what Artificial Intelligence can do. |
| Negative | 6 | I think artificially intelligent systems make many errors. |
| Positive | 7 | I am interested in using artificially intelligent systems in my daily life. |
| Negative | 8 | I find Artificial Intelligence sinister. |
| Negative | 9 | Artificial Intelligence might take control of people. |
| Negative | 10 | I think Artificial Intelligence is dangerous. |
| Positive | 11 | Artificial Intelligence can have positive impacts on people's wellbeing. |
| Positive | 12 | Artificial Intelligence is exciting. |
| **Attention Check** | **A** | I would be grateful if you could select agree. |
| Positive | 13 | An artificially intelligent agent would be better than an employee in many routine jobs. |
| Positive | 14 | There are many beneficial applications of Artificial Intelligence. |
| Negative | 15 | I shiver with discomfort when I think about future uses of Artificial Intelligence. |
| Positive | 16 | Artificially intelligent systems can perform better than humans. |
| Positive | 17 | Much of society will benefit from a future full of Artificial Intelligence |
| Positive | 18 | I would like to use Artificial Intelligence in my own job. |
| Negative | 19 | People like me will suffer if Artificial Intelligence is used more and more. |
| Negative | 20 | Artificial Intelligence is used to spy on people |

**GAAIS German version**

|  | Stimme voll zu | Stimme zu | neutral | Stimme nicht zu | Stimme überhaupt nicht zu |
| --- | --- | --- | --- | --- | --- |
| Für Routinearbeiten würde ich lieber mit einer KI zusammenarbeiten als mit einem Menschen. |  |  |  |  |  |
| KI kann uns neue ökonomische Möglichkeiten zur Verfügung stellen. |  |  |  |  |  |
| KI wird auf unethische Art und Weise genutzt. |  |  |  |  |  |
| KI-Programme können Menschen helfen glücklicher zu werden. |  |  |  |  |  |
| Ich bin beeindruckt, was KI kann. |  |  |  |  |  |
| Ich denke KI-Programme machen viele Fehler. |  |  |  |  |  |
| Ich interessiere mich dafür, KI in meinem täglichen Leben zu verwenden. |  |  |  |  |  |
| Ich finde KI unheimlich. |  |  |  |  |  |
| KI könnte die Kontrolle über die Menschheit gewinnen. |  |  |  |  |  |
| Ich glaube KI ist gefährlich. |  |  |  |  |  |
| KI kann einen positiven Einfluss auf das Wohlbefinden der Menschen haben. |  |  |  |  |  |
| KI ist aufregend. |  |  |  |  |  |
| Bitte machen Sie jetzt ein Kreuz bei „stimme zu“. |  |  |  |  |  |
| Ein KI-Programm ist besser als menschliche Mitarbeiter:innen in vielen Standardjobs. |  |  |  |  |  |
| Es gibt viele gute Möglichkeiten KI anzuwenden. |  |  |  |  |  |
| Beim Gedanken an eine Zukunft mit KI fühle ich mich sehr unwohl. |  |  |  |  |  |
| KI-Systeme leisten mehr als Menschen. |  |  |  |  |  |
| Der Großteil der Gesellschaft wird von einer Zukunft mit KI profitieren. |  |  |  |  |  |
| Ich würde gerne in meinem Beruf KI nutzen können. |  |  |  |  |  |
| Die Menschheit wird unter einer vermehrten KI-Nutzung leiden. |  |  |  |  |  |
| KI wird genutzt, um Menschen auszuspionieren. |  |  |  |  |  |

## State-Trait Anxiety Inventory (STAI)

### **Trait Anxiety**

| **Grimm-No.** | **Itemtext (german, Grimm)** | **Original English Item (Spielberger et al., 1970)** |
| --- | --- | --- |
| A01 | Ich werde schnell müde | *I tire quickly.* |
| A02 | Ich verpasse günstige Gelegenheiten, weil ich mich nicht schnell genug entscheiden kann | *I feel indecisive* |
| A03 | Ich bin ruhig und gelassen | *I am “calm, cool, and collected.”* |
| A04 | Ich bin glücklich | *I am happy.* |
| A05 | Ich neige dazu, alles schwer zu nehmen | *I take disappointments so keenly that I can’t put them out of my mind.* |
| A06 | Mir fehlt es an Selbstvertrauen | *I lack self-confidence.* |
| A07 | Ich fühle mich geborgen | *I feel secure.* |
| A08 | Ich fühle mich niedergeschlagen | *I feel like a failure.* |
| A09 | Unwichtige Gedanken gehen mir durch den Kopf und bedrücken mich | *Some unimportant thought runs through my mind and bothers me.* |
| A10 | Ich werde nervös und unruhig, wenn ich an meine derzeitigen Angelegenheiten denke | *I get in a state of tension or turmoil as I think over my recent concerns and interests.* |

### **State Anxiety**

| **Grimm-No.** | **Itemtext (german, Grimm)** | **Original English Item (Spielberger et al., 1970)** |
| --- | --- | --- |
| ASTA01 | Ich bin ruhig | *I feel calm.* |
| ASTA02 | Ich fühle mich angespannt | *I am tense.* |
| ASTA03 | Ich bin bekümmert | *I feel upset.* |
| ASTA04 | Ich fühle mich ausgeruht | *I feel at ease.* |
| ASTA05 | Ich bin beunruhigt | *I feel frightened.* |
| ASTA06 | Ich fühle mich selbstsicher | *I feel self-confident.* |
| ASTA07 | Ich bin nervös | *I feel nervous.* |
| ASTA08 | Ich bin verkrampft | *I feel strained.* |
| ASTA09 | Ich bin besorgt | *I am presently worrying over possible misfortunes.* |
| ASTA10 | Ich bin vergnügt | *I feel pleasant.* |
