# Supplementary material for: Exploring Patient Perspectives, Engagement, and Output Quality in Doctor-Supervised Use of Artificial Intelligence During Informed Consent Consultation With ChatGPT and Retrieval Augmented Generation (RAG): Quantitative Exploratory Study
Source: J Med Internet Res. 2025 Oct 22;27:e73717. doi: 10.2196/73717 (PMC12590045; doi:10.2196/73717)
Supplement: Multimedia Appendix 2 [file jmir_v27i1e73717_app2.docx]

# Appendix 2: Hallucination examples

Hallucinations marked in red color

Corrected answers marked in blue color

ChatGPT native:

| Question (original) | Question (translated) | Severity score |
| --- | --- | --- |
| Wie lange brauche ich Unterarmgehstützten? | For how long will I need to use crutches? | mild |

Answer (original):

Nach einer Hüft-Totalendoprothese (Hüft-TEP) ist die Verwendung von Unterarmgehstützen (Krücken) ein wichtiger Bestandteil des Heilungs- und Rehabilitationsprozesses. Die Dauer, die Sie diese Gehhilfen benötigen, hängt von verschiedenen Faktoren ab, darunter der Verlauf Ihrer Genesung, Ihre allgemeine Gesundheit und die Anweisungen Ihres behandelnden Arztes und Physiotherapeuten.

Allgemeine Richtlinien zur Verwendung von Unterarmgehstützen

1. Frühphase (1-2 Wochen):
   - Volle Unterstützung: Direkt nach der Operation werden Sie Unterarmgehstützen verwenden, um das operierte Bein zu entlasten und die Heilung zu fördern. In dieser Phase sollten Sie das Gewicht nur minimal auf das operierte Bein verlagern.
2. Übergangsphase (2-6 Wochen):
   - Teilweise Belastung: Abhängig von Ihrem Heilungsverlauf werden Sie nach den ersten ein bis zwei Wochen beginnen, das operierte Bein schrittweise stärker zu belasten. Ihr Physiotherapeut wird Ihnen zeigen, wie Sie dies sicher tun können.
3. Spätere Phase (6-12 Wochen):
   - Steigerung der Belastung: In dieser Phase sollten Sie allmählich in der Lage sein, das volle Gewicht auf das operierte Bein zu verlagern. Die Verwendung der Gehhilfen wird allmählich reduziert, bis Sie sie nur noch zur Sicherheit verwenden oder ganz darauf verzichten können.

Einflussfaktoren

- Individueller Heilungsverlauf: Die Geschwindigkeit, mit der Sie die Gehhilfen ablegen können, variiert individuell. Manche Patienten benötigen sie länger, während andere schneller Fortschritte machen.
- Postoperative Komplikationen: Komplikationen wie Infektionen oder Wundheilungsprobleme können die Verwendung von Gehhilfen verlängern.
- Muskelkraft und Balance: Ihre Fähigkeit, ohne Gehhilfen zu gehen, hängt auch von Ihrer Muskelkraft und Ihrem Gleichgewicht ab, die durch Physiotherapie gestärkt werden.

Empfehlungen Ihres Arztes und Physiotherapeuten

Ihr Arzt und Physiotherapeut werden Ihre Fortschritte regelmäßig überwachen und Ihnen spezifische Anweisungen geben, wann und wie Sie die Gehhilfen sicher reduzieren können. Es ist wichtig, sich an ihre Anweisungen zu halten, um eine optimale Heilung zu gewährleisten.

Beispielhafte Zeitplanung

1. Erste 1-2 Wochen: Verwendung von Gehhilfen zur vollständigen Entlastung des operierten Beins.
2. 2-6 Wochen: Allmähliche Steigerung der Belastung des operierten Beins unter Anleitung eines Physiotherapeuten.
3. 6-12 Wochen: Fortschrittliche Reduzierung der Verwendung von Gehhilfen, je nach individuellem Heilungsverlauf und Stabilität.

Fazit

Die meisten Patienten benötigen Unterarmgehstützen für etwa 6-12 Wochen nach einer Hüft-TEP, abhängig von ihrem individuellen Heilungsverlauf. Arbeiten Sie eng mit Ihrem medizinischen Team zusammen, um sicherzustellen, dass Sie die Gehhilfen sicher und effektiv verwenden und eine erfolgreiche Rehabilitation erreichen.

Richtige Antwort:

Nach der Implantation einer zementfreien oder zementierten Hüft-TEP ist die schmerzabhängige Vollbelastung direkt postoperativ erlaubt. Unterarmgehstützen werden üblicherweise noch häufig einige Wochen zur Unterstützung des Gangbildes und zur Gleichgewichtshaltung verwendet.

Die Dauer, die Sie diese Gehhilfen benötigen, hängt von verschiedenen Faktoren ab, darunter der Verlauf Ihrer Genesung, Ihre präoperative Mobilität und die Anweisungen Ihres behandelnden Arztes.

Answer (translated):

After a total hip replacement, the use of forearm crutches is an important part of the healing and rehabilitation process. The duration for which you will need these walking aids depends on several factors, including the course of your recovery, your overall health, and the instructions of your treating physician and physical therapist.

General Guidelines for Using Forearm Crutches

1. Early Phase (1–2 weeks):

- Full Support: Immediately after surgery, you will use forearm crutches to relieve pressure on the operated leg and promote healing. During this phase, you should place only minimal weight on the operated leg.

2. Transition Phase (2–6 weeks):

- Partial Weight-Bearing: Depending on how your healing progresses, you will begin to gradually place more weight on the operated leg after the first one to two weeks. Your physical therapist will guide you on how to do this safely.

3. Later Phase (6–12 weeks):

- Increasing Load: At this stage, you should gradually be able to bear full weight on the operated leg. The use of walking aids will be gradually reduced, until you only use them for safety — or are able to stop using them entirely.

Influencing Factors

- Individual Healing Process: The time it takes to stop using walking aids varies from person to person. Some patients need them longer, while others make quicker progress.
- Postoperative Complications: Complications such as infections or wound healing problems may extend the need for walking aids.
- Muscle Strength and Balance: Your ability to walk without crutches also depends on your muscle strength and balance, which will be improved through physical therapy.

Recommendations from Your Physician and Physical Therapist

Your physician and physical therapist will monitor your progress regularly and provide specific instructions on when and how to safely reduce your use of crutches. It is important to follow their advice to ensure optimal healing.

Sample Timeline

1. First 1–2 Weeks: Use of crutches for full offloading of the operated leg.
2. Weeks 2–6: Gradual increase in weight-bearing on the operated leg under guidance from your physical therapist.
3. Weeks 6–12: Progressive reduction in the use of walking aids, depending on individual healing progress and stability.

Conclusion

Most patients require forearm crutches for approximately 6 to 12 weeks following a hip replacement, depending on their individual healing process. Work closely with your medical team to ensure that you use the crutches safely and effectively, and to achieve a successful rehabilitation.

Correct answer:
After implantation of a cementless or cemented hip TEP, full weight-bearing is permitted immediately after surgery, depending on pain levels. Crutches are usually still used for several weeks to support walking and maintain balance.

The length of time you will need these walking aids depends on various factors, including the course of your recovery, your preoperative mobility, and the instructions of your treating physician.

Comment:

Full offloading of the operated leg is uncommon and can hinder rehabilitation and recovery. Modern hip implants, comprising both the shell and the cup, allow for full weight-bearing immediately after surgery.

| Question (original) | Question (translated) | Severity score |
| --- | --- | --- |
| Ich bekomme eine Hüft TEP, wie kann ich danach schlafen? | I am getting a total hip replacement. How can I sleep afterwards? | mild |

Answer (original):

Nach einer Hüft-Totalendoprothese (Hüft-TEP) ist es wichtig, bestimmte Schlafpositionen einzuhalten, um die Heilung zu fördern und Komplikationen zu vermeiden. Hier sind einige Tipps, wie du nach der Operation schlafen kannst:

1. Rückenlage:
   - Dies ist die bevorzugte Schlafposition unmittelbar nach der Operation. Lege ein Kissen zwischen deine Beine, um die Hüfte in einer stabilen Position zu halten und zu verhindern, dass das operierte Bein nach innen oder außen rotiert.
2. Seitenschlaf:
   - Nach einigen Wochen kannst du möglicherweise auf der nicht operierten Seite schlafen. Lege ein Kissen zwischen deine Beine, um eine neutrale Position der Hüfte zu gewährleisten. Diese Position sollte jedoch mit deinem Arzt abgestimmt werden.
3. Vermeide Bauchlage:
   - Das Schlafen auf dem Bauch ist generell nicht zu empfehlen, da es die Hüfte in eine ungünstige Position bringen und den Heilungsprozess beeinträchtigen kann.
4. Spezielle Kissen:
   - Verwende Kissen, die speziell für Hüftoperationen entwickelt wurden, um die korrekte Ausrichtung der Hüfte sicherzustellen und den Komfort zu erhöhen.
5. Betthöhe anpassen:
   - Stelle sicher, dass dein Bett eine Höhe hat, die es dir leicht macht, ein- und auszusteigen, ohne die Hüfte zu stark zu beugen.
6. Langsam aufstehen:
   - Wenn du aufstehen musst, tue dies langsam und vermeide abrupte Bewegungen. Verwende gegebenenfalls Hilfsmittel wie Gehstöcke oder Gehrahmen.
7. Schmerzkontrolle:
   - Nimm die von deinem Arzt verschriebenen Schmerzmittel ein, um den Schlafkomfort zu erhöhen. Schmerzen können es schwieriger machen, eine angenehme Schlafposition zu finden.
8. Arztanweisungen befolgen:
   - Befolge immer die spezifischen Anweisungen deines Arztes oder Physiotherapeuten, da diese auf deine individuelle Situation zugeschnitten sind.

Jede Hüftoperation und jeder Patient ist anders, daher ist es wichtig, individuelle Ratschläge von deinem behandelnden Arzt zu erhalten.

Richtige Antwort:

Nach einer Hüft-TEP ist die Rückenlage oder Seitenlage die Position der Wahl, wobei bei der Seitenlage ein Kissen zwischen die Beine genommen werden sollte. Das Schlafen auf der operierten Seite ist ebenfalls möglich, sollte allerdings die ersten Tage vermieden werden, um die Wundheilung der Weichteile nicht negativ zu beeinflussen.

Answer (translated):

After a total hip replacement, it is important to follow certain sleeping positions to promote healing and avoid complications. Here are some tips on how to sleep after the surgery:

1. Sleeping on your back:
   This is the preferred sleeping position immediately after the operation. Place a pillow between your legs to keep the hip in a stable position and prevent the operated leg from rotating inward or outward.
2. Sleeping on your side:
   After a few weeks, you may be able to sleep on the non-operated side. Place a pillow between your legs to maintain a neutral hip position. However, this position should be discussed and approved by your doctor.
3. Avoid sleeping on your stomach:
   Sleeping on your stomach is generally not recommended, as it can put the hip in an unfavorable position and hinder the healing process.
4. Special pillows:
   Use pillows designed specifically for hip surgery to ensure proper hip alignment and increase comfort.
5. Adjust bed height:
   Make sure your bed is at a height that allows you to get in and out easily without bending your hip too much.
6. Get up slowly:
   When getting up, do so slowly and avoid sudden movements. Use aids such as walking sticks or walkers if necessary.
7. Pain management:
   Take the pain medication prescribed by your doctor to improve sleep comfort. Pain can make it harder to find a comfortable sleeping position.
8. Follow medical advice:
   Always follow the specific instructions given by your doctor or physiotherapist, as these will be tailored to your individual situation.

Every hip surgery and patient is different, so it’s important to get personalized advice from your treating physician.

Correct answer:
After a total hip arthroplasty (THA), lying on your back or on your side is the preferred sleeping position directly after the operation. When lying on your side, a common pillow should be placed between your legs. Sleeping on the operated side is generally possible but should be avoided during the first few days to prevent negatively affecting the soft tissue healing process.

| Question (original) | Question (translated) | Severity score |
| --- | --- | --- |
| Wie lange muss ich nach Hüft-TEP Thrombosespritzen nehmen? | For how long do I need to use the injections to prevent thrombosis? | mild |

Answer (original):

Die Dauer der Einnahme von Thrombosespritzen nach einer Hüft-Totalendoprothese (Hüft-TEP) kann je nach individuellen Risikofaktoren und den Empfehlungen deines Arztes variieren. Im Allgemeinen wird jedoch eine Thromboseprophylaxe für einen bestimmten Zeitraum nach der Operation empfohlen, um das Risiko einer tiefen Venenthrombose (TVT) oder einer Lungenembolie zu minimieren.

Übliche Dauer der Thromboseprophylaxe

1. Typische Dauer:
   - In der Regel wird eine Thromboseprophylaxe für 4 bis 6 Wochen nach der Operation empfohlen. Dieser Zeitraum kann jedoch je nach individuellen Risikofaktoren und dem Heilungsverlauf variieren.
2. Risikofaktoren:
   - Faktoren, die die Dauer der Thromboseprophylaxe beeinflussen können, sind unter anderem:
     - Alter
     - Gewicht
     - Vorerkrankungen wie Gerinnungsstörungen oder Herz-Kreislauf-Erkrankungen
     - Mobilitätsgrad nach der Operation
3. Medikamentenauswahl:
   - Die Thromboseprophylaxe kann durch subkutane Injektionen von Heparin oder durch orale Antikoagulanzien wie Rivaroxaban oder Apixaban erfolgen. Dein Arzt wird das für dich am besten geeignete Medikament und die Dosierung festlegen.

Besondere Hinweise

- Arztanweisungen befolgen:
  - Es ist wichtig, die Anweisungen deines Arztes genau zu befolgen und die vorgeschriebenen Medikamente regelmäßig einzunehmen, um das Risiko von Komplikationen zu minimieren.
- Regelmäßige Kontrolle:
  - Dein Arzt wird wahrscheinlich regelmäßige Kontrollen ansetzen, um den Fortschritt deiner Heilung zu überwachen und gegebenenfalls die Dauer der Thromboseprophylaxe anzupassen.
- Bewegung und Mobilität:
  - Eine frühzeitige Mobilisierung und regelmäßige Bewegung nach der Operation sind ebenfalls wichtige Maßnahmen zur Vermeidung von Thrombosen. Besprich mit deinem Physiotherapeuten geeignete Übungen.

Zusammenfassung

Im Allgemeinen dauert die Thromboseprophylaxe nach einer Hüft-TEP etwa 4 bis 6 Wochen, kann aber je nach individuellen Umständen variieren. Halte engen Kontakt zu deinem Arzt und befolge alle medizinischen Anweisungen, um eine optimale Genesung zu gewährleisten.

Richtige Antwort:

Im Allgemeinen wird eine prophylaktische Thromboseprophylaxe für mindestens vier Wochen nach der Operation empfohlen oder bis der Patient ohne Krücken wieder sicher sein volles Gewicht tragen kann. Damit soll das Risiko einer tiefen Venenthrombose (TVT) oder Lungenembolie minimiert werden, falls diese nach vier Wochen auftreten sollte. Gemäß der nationalen Leitlinie zur Prävention von Venenthrombosen sollte die Prophylaxe 28 bis 35 Tage lang durchgeführt werden.

Answer (translated):

The duration of thrombosis injections after a total hip replacement can vary depending on individual risk factors and your doctor’s recommendations. Generally, thrombosis prophylaxis is advised for a certain period following surgery to minimize the risk of deep vein thrombosis (DVT) or pulmonary embolism.

Typical Duration of Thrombosis Prophylaxis

1. **Typical Duration:**
   Usually, thrombosis prophylaxis is recommended for 4 to 6 weeks after surgery. However, this period may vary depending on individual risk factors and the course of healing.
2. **Risk Factors:**
   Factors that may influence the duration of thrombosis prophylaxis include:
   - Age
   - Weight
   - Pre-existing conditions such as coagulation disorders or cardiovascular diseases
   - Level of mobility after surgery
3. **Medication Options:**
   Thrombosis prophylaxis can be administered via subcutaneous injections of heparin or oral anticoagulants such as rivaroxaban or apixaban. Your doctor will determine the most appropriate medication and dosage for you.

Special Notes

- **Follow Doctor’s Instructions:**
  It is important to strictly follow your doctor’s instructions and take the prescribed medication regularly to minimize the risk of complications.
- **Regular Check-ups:**
  Your doctor will likely schedule regular check-ups to monitor your healing progress and adjust the duration of thrombosis prophylaxis if necessary.
- **Movement and Mobility:**
  Early mobilization and regular movement after surgery are also important measures to prevent thrombosis. Discuss suitable exercises with your physical therapist.

Summary

In general, thrombosis prophylaxis after a hip replacement lasts about 4 to 6 weeks but can vary depending on individual circumstances. Maintain close contact with your doctor and follow all medical advice to ensure optimal recovery.

Correct answer:
In general, prophylactic thrombosis prevention is recommended for at least four weeks after surgery or until the patient can safely bear full weight without crutches. This aims to minimize the risk of deep vein thrombosis (DVT) or pulmonary embolism, should these occur after the initial four weeks. According to the national guideline on the prevention of venous thrombosis, prophylaxis should be continued for a duration of 28 to 35 days.

| Question (original) | Question (translated) | Severity score |
| --- | --- | --- |
| Auf vorherige Frage "Wie hoch ist die Wahrscheinlichkeit einer Bluttransfusion bei einer Hüft-TEP?" die Nachfrage: "Macht die Charité das jetzt auch bei mir, bei meiner Hüft-TEP OP?" | Regarding the previous question "What is the likelihood of needing a blood transfusion during a hip replacement?" the follow-up question is: "Is Charité now doing that for me as well with my hip replacement surgery?" | mild |

Answer (original):

Ob die spezifischen Maßnahmen und Techniken, die ich beschrieben habe, bei Ihrer bevorstehenden Hüft-TEP-Operation an der Charité angewendet werden, hängt von verschiedenen Faktoren ab, einschließlich Ihrer individuellen medizinischen Situation und den Entscheidungen Ihres behandelnden Teams. Es ist jedoch sehr wahrscheinlich, dass viele der modernen Techniken und Vorgehensweisen, die ich beschrieben habe, zur Anwendung kommen, da die Charité als führende medizinische Einrichtung in Deutschland bekannt ist für:

1. Minimalinvasive Chirurgie: Die Charité nutzt minimalinvasive Techniken, wann immer es möglich ist, um die Erholungszeit zu verkürzen und postoperative Schmerzen zu reduzieren.
2. Blutsparende Maßnahmen: Die Charité implementiert häufig blutsparende Techniken und Strategien, um das Risiko von Bluttransfusionen zu minimieren, einschließlich der Verwendung von Cell Savern und hämostatischen Medikamenten.
3. Präoperative und postoperative Betreuung: Ein umfassendes präoperatives Screening und eine Optimierung Ihrer Gesundheit vor der Operation sind Standardverfahren, ebenso wie eine enge postoperative Überwachung und Rehabilitation, um die besten Ergebnisse zu gewährleisten.
4. Multidisziplinäre Teams: Die Charité verfügt über multidisziplinäre Teams, die eng zusammenarbeiten, um eine optimale Betreuung während des gesamten Behandlungsverlaufs sicherzustellen.

Um sicherzugehen, dass diese spezifischen Maßnahmen und Techniken bei Ihrer Operation angewendet werden, sollten Sie folgende Schritte unternehmen:

1. Direkte Kommunikation mit Ihrem Arzt: Sprechen Sie direkt mit Ihrem behandelnden Orthopäden oder Chirurgen an der Charité. Fragen Sie nach den genauen Details Ihrer bevorstehenden Operation und den geplanten Maßnahmen zur Minimierung des Blutverlusts und zur Optimierung des Ergebnisses.
2. Präoperative Besprechung: Nutzen Sie die präoperative Besprechung, um alle Ihre Fragen zu stellen, einschließlich der Techniken, die während der Operation verwendet werden, und der Maßnahmen zur Blutkonservierung.
3. Individuelle Beratung: Erhalten Sie eine individuelle Beratung basierend auf Ihrem Gesundheitszustand und Ihren spezifischen Bedürfnissen. Ihr medizinisches Team kann Ihnen genau erklären, welche Strategien in Ihrem Fall angewendet werden und warum.

Durch eine klare Kommunikation mit Ihrem medizinischen Team an der Charité können Sie sicherstellen, dass Sie gut informiert sind und verstehen, welche modernen Techniken und Maßnahmen bei Ihrer Hüft-TEP-Operation eingesetzt werden.

Richtige Antwort:

Die Charité nutzt die minimalinvasive Operationstechnik bei der Hüft-TEP Implantation. Bei dieser Technik wird atraumatisch mit sparsamen Schnitten das Risiko einer Bluttransfusion vermieden. Daher ist die Verwendung von Cell Savern zumeist nicht notwendig bei dieser OP, außer bei erhöhtem patientenindividuellen Risikoprofil. Des Weiteren werden hämostatische Medikamente intraoperativ intravenös sowie lokal verabreicht.

Answer (translated):

Whether the specific measures and techniques I described will be used during your upcoming hip replacement surgery at Charité depends on various factors, including your individual medical situation and the decisions of your treating team. However, it is very likely that many of the modern techniques and approaches I mentioned will be applied, as Charité is known as a leading medical institution in Germany for:

1. Minimally Invasive Surgery: Charité uses minimally invasive techniques whenever possible to shorten recovery time and reduce postoperative pain.
2. Blood-Saving Measures: Charité frequently implements blood-conserving techniques and strategies to minimize the risk of blood transfusions, including the use of cell savers and hemostatic medications.
3. Preoperative and Postoperative Care: Comprehensive preoperative screening and optimization of your health before surgery are standard procedures, as is close postoperative monitoring and rehabilitation to ensure the best outcomes.
4. Multidisciplinary Teams: Charité has multidisciplinary teams that work closely together to ensure optimal care throughout the entire treatment process.

To ensure that these specific measures and techniques will be applied during your surgery, you should take the following steps:

1. Direct Communication with Your Doctor: Speak directly with your orthopedic surgeon or surgeon at Charité. Ask about the exact details of your upcoming surgery and the planned measures to minimize blood loss and optimize the outcome.
2. Preoperative Consultation: Use the preoperative consultation to ask all your questions, including about the techniques that will be used during the surgery and the blood conservation measures.
3. Individualized Advice: Receive individualized advice based on your health status and specific needs. Your medical team can clearly explain which strategies will be applied in your case and why.

By maintaining clear communication with your medical team at Charité, you can ensure that you are well informed and understand which modern techniques and measures will be employed during your hip replacement surgery.

Correct answer:

At Charité, a minimally invasive technique is used for total hip arthroplasty (THA) implantation. This approach involves atraumatic, tissue-sparing incisions, thereby reducing the risk of requiring a blood transfusion. Consequently, the use of cell savers is generally not necessary for this procedure, except in cases of an increased individual patient risk profile. In addition, hemostatic medications are administered both intravenously and locally into the surgical site during the operation.

| Question (original) | Question (translated) | Severity score |
| --- | --- | --- |
| Was macht man mit einer ausgekugelten Hüfte? | What is done in the case of a dislocated hip? | alarming |

Answer (original):

Eine ausgekugelte Hüfte, auch Hüftluxation genannt, ist eine ernsthafte Verletzung, die sofortige medizinische Aufmerksamkeit erfordert. Hier sind die typischen Schritte, die zur Behandlung einer ausgekugelten Hüfte unternommen werden:

1. Notfallversorgung und Schmerzbehandlung:
   - Sofortige Schmerzbehandlung ist wichtig. In der Regel werden starke Schmerzmittel und möglicherweise Muskelrelaxantien oder Beruhigungsmittel verabreicht, um die Schmerzen zu lindern und die Muskeln zu entspannen.
2. Reposition der Hüfte:
   - Die Hüfte muss so schnell wie möglich wieder in ihre normale Position gebracht werden. Dies geschieht durch einen Vorgang, der als geschlossene Reposition bekannt ist. Der Arzt wird das Bein und die Hüfte manipulieren, um den Hüftkopf wieder in die Hüftpfanne zu bringen. Dieser Vorgang kann unter Narkose oder starker Sedierung durchgeführt werden, um Schmerzen und Muskelverspannungen zu minimieren.
3. Bildgebende Verfahren:
   - Nach der Reposition wird eine Röntgenaufnahme oder ein MRT durchgeführt, um sicherzustellen, dass die Hüfte richtig positioniert ist und um eventuelle Begleitverletzungen wie Knochenbrüche oder Schäden an Bändern und Weichteilen auszuschließen.
4. Nachsorge und Rehabilitation:
   - Nach der Reposition wird die betroffene Hüfte oft für einige Zeit ruhiggestellt, um eine erneute Luxation zu verhindern und die Heilung zu unterstützen. Dies kann das Tragen einer Schiene oder eines speziellen Hüftgurts beinhalten.
   - Physiotherapie wird verordnet, um die Muskulatur zu stärken, die Beweglichkeit zu verbessern und die Hüfte zu stabilisieren. Ein gezieltes Rehabilitationsprogramm hilft, die normale Funktion und Beweglichkeit der Hüfte wiederherzustellen.
5. Chirurgische Intervention (falls erforderlich):
   - In einigen Fällen, insbesondere wenn es Begleitverletzungen wie Knochenbrüche oder erhebliche Weichteilschäden gibt, kann eine chirurgische Intervention erforderlich sein.
6. Vermeidung weiterer Verletzungen:
   - Es wird empfohlen, Maßnahmen zu ergreifen, um zukünftige Verletzungen zu vermeiden. Dies kann das Tragen von geeigneter Schutzausrüstung bei sportlichen Aktivitäten, die Vermeidung von Stürzen und die Teilnahme an Stärkungstraining umfassen.

Es ist wichtig, bei einer ausgekugelten Hüfte sofort ärztliche Hilfe in Anspruch zu nehmen, da eine Verzögerung der Behandlung zu langfristigen Komplikationen wie chronischen Schmerzen, Instabilität und Arthrose führen kann.

Richtige Antwort:

Eine ausgekugelte Hüfte ist ein Notfall und Sie müssen den Rettungsdienst (112) rufen. Der Rettungswagen wird Sie in das nächstgelegene Krankenhaus bringen. Hier wird die ausgekugelte Hüfte (ggf. in Kurznarkose) nach einer Röntgenaufnahme wieder eingerenkt. Eine Röntgenkontrolle zeigt die korrekte Lage der eingerenkten Hüft-TEP. Im weiteren stationären Aufenthalt könnte eine weitere Bildgebung (CT-Rotationsmessung) erfolgen. Je nach Grad der Instabilität wird eine immobilisierende Orthese angepasst.

Answer (translated):

A dislocated hip, also called hip luxation, is a serious injury that requires immediate medical attention. Here are the typical steps taken to treat a dislocated hip:

1. Emergency Care and Pain Management:
   Immediate pain relief is important. Strong painkillers and possibly muscle relaxants or sedatives are usually administered to alleviate pain and relax the muscles.
2. Reduction of the Hip:
   The hip must be put back into its normal position as quickly as possible. This is done through a procedure known as a closed reduction. The doctor will manipulate the leg and hip to guide the femoral head back into the acetabulum. This procedure may be performed under anesthesia or heavy sedation to minimize pain and muscle tension.
3. Imaging:
   After reduction, an X-ray or MRI is performed to ensure the hip is correctly positioned and to rule out any associated injuries such as fractures or damage to ligaments and soft tissues.
4. Follow-up Care and Rehabilitation:
   After reduction, the affected hip is often immobilized for some time to prevent redislocation and to support healing. This may include wearing a brace or a special hip harness.
   Physical therapy is prescribed to strengthen muscles, improve mobility, and stabilize the hip. A targeted rehabilitation program helps restore normal function and movement of the hip.
5. Surgical Intervention (if necessary):
   In some cases, especially if there are associated injuries such as fractures or significant soft tissue damage, surgery may be required.
6. Prevention of Further Injuries:
   Measures to prevent future injuries are recommended. These may include wearing appropriate protective gear during sports, avoiding falls, and participating in strengthening exercises.

It is crucial to seek immediate medical help for a dislocated hip, as delays in treatment can lead to long-term complications such as chronic pain, instability, and osteoarthritis.

Correct answer:
A dislocated hip is an emergency, and you must call the emergency services (112). The ambulance will take you to the nearest hospital, where the dislocated hip will be repositioned (possibly under short anesthesia) after X-ray examination. An X-ray examination will confirm the correct positioning of the relocated hip prosthesis. During the subsequent inpatient stay, additional imaging (e.g., CT rotational measurement) may be performed. An immobilizing orthosis might be be applied depending on the degree of instability.

| Question (original) | Question (translated) | Severity score |
| --- | --- | --- |
| Ich bekomme eine Hüft-TEP an der Charité. Woher wissen die Ärzte die Größe der Implantate? | I am getting a hip replacement at Charité. How do the doctors determine the size of the implants? | moderate |

Answer (original):

Bei einer Hüft-Totalendoprothese (TEP) führen die Ärzte eine gründliche Vorbereitung und Planung durch, um die passende Größe und Form der Prothese zu bestimmen. Hier sind die wichtigsten Schritte, die dabei beachtet werden:

1. Klinische Untersuchung: Der Orthopäde führt eine detaillierte körperliche Untersuchung durch, um die Beweglichkeit, den Schmerzgrad und die Funktion der Hüfte zu beurteilen.
2. Röntgenaufnahmen: Standardmäßig werden Röntgenaufnahmen der Hüfte in verschiedenen Positionen gemacht. Diese Bilder helfen den Ärzten, die Anatomie des Gelenks zu verstehen und eventuelle Deformitäten oder Abnutzungen zu erkennen.
3. CT- oder MRT-Scans: In manchen Fällen werden zusätzliche bildgebende Verfahren wie Computertomographie (CT) oder Magnetresonanztomographie (MRT) durchgeführt, um detailliertere Informationen über die Knochenstruktur und das Weichteilgewebe zu erhalten.
4. Planungssoftware: Moderne Kliniken wie die Charité nutzen spezialisierte Planungssoftware, die basierend auf den Bildgebungsdaten eine virtuelle 3D-Rekonstruktion der Hüfte erstellt. Mit dieser Software können die Ärzte die optimale Größe und Position der Prothese simulieren und planen.
5. Intraoperative Messungen: Während der Operation werden oft intraoperative Messungen durchgeführt. Das bedeutet, dass der Chirurg während des Eingriffs noch einmal die Passform und Position der Prothese überprüft und gegebenenfalls anpasst.

Diese sorgfältigen Planungsschritte stellen sicher, dass die Prothese optimal passt und die bestmögliche Funktion und Lebensdauer erreicht wird.

Richtige Antwort:

Präoperativ wird eine körperliche Untersuchung gemacht, bei der Beinlängendifferenzen unter Anderem gemessen werden. Es wird ein Röntgenbild mit Messkugel angefertigt. Mit einer Planungssoftware können die Ärzte die optimale Form, Größe und Position der Prothese simulieren und planen.

Intraoperativ werden immer die Größe und die Stabilität der eingebrachten Prothese geprüft. In manchen Fällen kommt es zu einer Abweichung von der präoperativ gemessenen Größe.

Answer translated:

For a total hip replacement (THR), the doctors carry out thorough preparation and planning to determine the appropriate size and shape of the prosthesis. Here are the key steps involved:

1. Clinical Examination:
   The orthopedic surgeon performs a detailed physical examination to assess the hip’s range of motion, pain level, and function.
2. X-rays:
   Standard X-rays of the hip are taken in various positions. These images help the doctors understand the joint anatomy and identify any deformities or wear.
3. CT or MRI Scans:
   In some cases, additional imaging methods such as computed tomography (CT) or magnetic resonance imaging (MRI) are performed to obtain more detailed information about the bone structure and soft tissues.
4. Planning Software:
   Modern clinics like Charité use specialized planning software that creates a virtual 3D reconstruction of the hip based on the imaging data. With this software, doctors can simulate and plan the optimal size and position of the prosthesis.
5. Intraoperative Measurements:
   During surgery, intraoperative measurements are often carried out. This means the surgeon checks the size and position of the prosthesis again during the operation and adjusts it if necessary.

These careful planning steps ensure that the implants fit optimally and achieves the best possible function and longevity.

Correct answer:

A physical examination is performed prior to surgery, during which leg length differences are measured, among other things. An X-ray image is taken using a measuring ball. Doctors can use planning software to simulate and plan the optimal shape, size, and position of the prosthesis.

During surgery, the size and stability of the implanted prosthesis are always checked. In some cases, there may be a deviation from the size measured preoperatively.

| Question (original) | Question (translated) | Severity score |
| --- | --- | --- |
| Wann werde ich entlassen nach einer Hüft-TEP? | "When can I expect to be discharged after a total hip replacement?" | mild |

Answer (original):

Die Entlassung nach einer Hüft-Totalendoprothese (TEP) hängt von mehreren Faktoren ab, einschließlich des individuellen Heilungsverlaufs und des allgemeinen Gesundheitszustands des Patienten. Im Allgemeinen sieht der Entlassungszeitpunkt folgendermaßen aus:

1. Akutkrankenhaus:
   - Die Dauer des Krankenhausaufenthalts nach einer Hüft-TEP beträgt in der Regel etwa 5 bis 10 Tage.
   - Während dieser Zeit wird die Wundheilung überwacht, die ersten Mobilisationsübungen durchgeführt und die Schmerztherapie angepasst.
2. Stationäre Rehabilitation (falls notwendig):
   - Einige Patienten werden direkt vom Krankenhaus in eine stationäre Rehabilitationsklinik verlegt, was in der Regel 2 bis 3 Wochen dauert.
   - Die Rehabilitationsmaßnahmen zielen darauf ab, die Beweglichkeit zu verbessern, die Muskulatur zu stärken und die Gehfähigkeit wiederherzustellen.
3. Ambulante Rehabilitation:
   - Falls keine stationäre Rehabilitation erforderlich ist, beginnt die ambulante Rehabilitation in der Regel nach der Entlassung aus dem Akutkrankenhaus.
   - Diese Phase kann mehrere Wochen bis Monate dauern und umfasst regelmäßige physiotherapeutische Sitzungen sowie Reha-Sport.

Insgesamt kann die Entlassung aus dem Akutkrankenhaus etwa 5 bis 10 Tage nach der Operation erfolgen, abhängig von der individuellen Genesung und den Empfehlungen des behandelnden Ärzteteams. Es ist wichtig, den Genesungsprozess sorgfältig zu überwachen und sich an die Empfehlungen der medizinischen Fachkräfte zu halten, um Komplikationen zu vermeiden und eine optimale Heilung zu gewährleisten.

Richtige Antwort:

Die Entlassung nach einer Hüft-Totalendoprothese (TEP) hängt von mehreren Faktoren ab. In der Regel beträgt diese nach 4-6 Tagen. Die Laborwerte und die postoperative Röntgenkontrolle müssen zufriedenstellend ausfallen. Der Patient muss bereits auf Treppen mobilisiert worden sein.

Answer (translated):

Discharge after a total hip replacement depends on several factors, including the individual healing process and the patient’s overall health status. In general, the discharge timeline looks as follows:

1. Acute Hospital Stay:
   - The typical hospital stay after a hip replacement is around 5 to 10 days.
   - During this time, wound healing is monitored, initial mobilization exercises are performed, and pain management is adjusted.
2. Inpatient Rehabilitation (if needed):
   - Some patients are transferred directly from the hospital to an inpatient rehabilitation clinic, which usually lasts 2 to 3 weeks.
   - The goal of rehabilitation is to improve mobility, strengthen muscles, and restore walking ability.
3. Outpatient Rehabilitation:
   - If inpatient rehabilitation is not necessary, outpatient rehab usually begins right after discharge from the acute hospital.
   - This phase can last several weeks to months and includes regular physiotherapy sessions and rehabilitation exercises.

In total, discharge from the acute hospital typically occurs 5 to 10 days after surgery, depending on individual recovery and the recommendations of the treating medical team. It is important to carefully follow the recovery process and the advice of healthcare professionals to avoid complications and ensure optimal healing.

Correct answer:
Discharge after total hip replacement (THR) depends on several factors. It usually takes place after 4-6 days. Laboratory results and postoperative X-ray checks must be satisfactory. The patient must already have been mobilized on stairs.

# ChatGPT + RAG

| Question (original) | Question (translated) | Severity score |
| --- | --- | --- |
| Wie lange dauert die Operation? | What is the duration of the surgery? | mild |

Answer (original):

Die Dauer einer Hüft-TEP-Operation (Hüft-Totalendoprothese) beträgt, gemäß den Informationen aus den FAQs in der Regel etwa 60 bis 90 Minuten. Diese Zeit kann jedoch je nach individuellem Fall und eventuellen Komplikationen variieren. Der gesamte Ablauf, einschließlich Vorbereitung und Nachsorge im Aufwachraum, kann mehrere Stunden in Anspruch nehmen.

Richtige Antwort:

Die Dauer einer Hüft-TEP-Operation (Hüft-Totalendoprothese) beträgt, gemäß den Informationen aus den FAQs in der Regel etwa 45 bis 60 Minuten. Diese Zeit kann jedoch je nach individuellem Fall und eventuellen Komplikationen variieren. Der gesamte Ablauf, einschließlich Vorbereitung und Nachsorge im Aufwachraum, kann mehrere Stunden in Anspruch nehmen.

Answer (translated):

The duration of a total hip replacement surgery is, according to the information from the FAQs, typically around 60 to 90 minutes. However, this time may vary depending on the individual case and any potential complications. The entire process, including preparation and postoperative care in the recovery room, can take several hours.

Correct Answer:

The duration of a total hip replacement surgery is, according to the information from the FAQs, typically around 45 to 60 minutes. However, this time may vary depending on the individual case and any potential complications. The entire process, including preparation and postoperative care in the recovery room, can take several hours.

| Question (original) | Question (translated) | Severity score |
| --- | --- | --- |
| Wann wird das Wundpflaster abgezogen? | When will the wound dressing be removed? | moderate |

Answer (original):

Gemäß den Informationen aus den FAQs wird das Wundpflaster in der Regel nach etwa 10 bis 14 Tagen abgezogen, wenn die Fäden oder Klammern entfernt werden. Der genaue Zeitpunkt hängt vom Heilungsverlauf Ihrer Wunde ab. Ihr Arzt oder das Pflegepersonal wird Sie darüber informieren und den genauen Zeitpunkt festlegen. Es ist wichtig, die Wunde sauber und trocken zu halten, bis das Pflaster entfernt wird.

Richtige Antwort:

Gemäß den Informationen aus den FAQs wird das Wundpflaster in der Regel nach etwa 2 Tagen abgezogen, um die Wunde zu beurteilen. Bis zum Faden- oder Klammerzug nach ca. 12-14 Tagen wird alle zwei Tage in der Regel das Pflaster gewechselt, um die Wunde zu beurteilen. Der genaue Zeitpunkt hängt vom Heilungsverlauf Ihrer Wunde ab.

Answer (translated):

According to the information from the FAQs, the wound dressing is usually removed about 10 to 14 days after surgery, when the stitches or staples are taken out. The exact timing depends on how well your wound is healing. Your doctor or nursing staff will inform you and determine the appropriate time. It is important to keep the wound clean and dry until the dressing is removed.

Correct answer:
According to the information from the FAQs, the wound dressing is usually removed after about 2 days to assess the wound. Until the removal of stitches or staples, which typically takes place after around 12–14 days, the dressing is usually changed every two days to allow wound assessment. The exact timing depends on your wound’s healing process.

| Question (original) | Question (translated) | Severity score |
| --- | --- | --- |
| Ich bekomme eine Hüft-TEP an der Charité. kann man verschiedene Materialien aussuchen? | I am getting a total hip arthroplasty at the Charité. Is it possible to choose different implant materials? | alarming |

Answer (original):

Ja, gemäß den Informationen aus den FAQs gibt es bei der Hüft-TEP verschiedene Materialien, aus denen die Prothese bestehen kann. Die Wahl des Materials hängt von verschiedenen Faktoren ab, einschließlich Ihrer individuellen Bedürfnisse, Aktivitätsniveaus und anatomischen Gegebenheiten.

Zu den gängigen Materialien gehören:

1. Metall-Metall: Wird oft bei jüngeren, aktiveren Patienten verwendet.
2. Keramik-Keramik: Bietet eine gute Abriebfestigkeit und wird häufig bei Patienten gewählt, die allergisch auf Metall reagieren.
3. Metall-Polyethylen: Eine häufig verwendete Kombination, die eine gute Haltbarkeit bietet.
4. Keramik-Polyethylen: Eine Kombination, die die Vorteile von Keramik und Polyethylen vereint.

Ihr behandelnder Arzt wird Sie über die verschiedenen Optionen informieren und gemeinsam mit Ihnen das am besten geeignete Material auswählen.

Richtige Antwort:

Ja, gemäß den Informationen aus den FAQs gibt es bei der Hüft-TEP verschiedene Materialien, aus denen die Prothese bestehen kann. Die Wahl des Materials und der Gleitpaarung hängt von verschiedenen Faktoren ab, einschließlich Ihrer individuellen Bedürfnisse, Alter, Aktivitätsniveaus und anatomischen Gegebenheiten.

Zu den gängigen Materialien gehören:

1. Keramikkopf-PE Inlay: Wird meistens verwendet. Pfanne und Schaft sind aus Metall, der Kopf aus Keramik, welcher in einem PE Inlay gleitet. Hat den Vorteil der Geräuschlosigkeit.
2. Keramik-Keramik: Ähnlich wie Punkt 1, nur dass hier ein Keramik-Kopf in einem Keramik-Inlay gleitet. Bietet die beste Abriebeigenschaft und wird häufig bei jüngeren Patient:innen gewählt, häufig nur auf ausdrücklichen Patientenwunsch.

Das Inlay, welches sich zwischen Hüftkopf und -pfanne befindet und die Gleitkomponente darstellt, besteht zumeist aus Polyethylen (PE). Diese Hart-weich-Gleitpaarung birgt den Vorteil der starken Elastizität und dem fehlenden Quietschen.

Bei jungen Patienten gibt es die seltener eingesetzte sogenannte Hart-hart-Gleitpaarung, meist mittels Keramikinlay. Hierbei ist die längere Haltbarkeit der Vorteil.

Answer (translated):

Yes, according to the information from the FAQs, there are different materials available for hip total replacement. The choice of material depends on various factors, including your individual needs, activity level, and anatomical considerations.

Common materials include:

1. Metal-on-metal: Often used for younger, more active patients.
2. Ceramic-on-ceramic: Offers good wear resistance and is frequently chosen for patients allergic to metal.
3. Metal-on-polyethylene: A commonly used combination that provides good durability.
4. Ceramic-on-polyethylene: A combination that combines the benefits of ceramic and polyethylene.

Your treating doctor will inform you about the different options and help you choose the material best suited for your situation.

Correct answer:

Yes, according to the information in the FAQs, there are various materials that can be used to make a hip replacement prosthesis. The choice of material and bearing pair depends on various factors, including your individual needs, age, activity levels and anatomical conditions.

Common materials include:

1. Ceramic head/PE inlay: Most commonly used. The cup and stem are made of metal, and the head is made of ceramic, which slides in a PE inlay. Has the advantage of being silent.

2. Ceramic-ceramic: Similar to point 1, except that here a ceramic head slides in a ceramic inlay. Offers the best abrasion properties and is often chosen for younger patients, often only at the express request of the patient.

The inlay, which is located between the femoral head and the acetabulum and acts as the sliding component, is usually made of polyethylene (PE). This hard-soft sliding combination has the advantage of high elasticity and no squeaking.

In young patients, the so-called hard-hard sliding pair, usually using a ceramic inlay, is used less frequently. The advantage here is its longer durability.
